# Novel heavy metal resistance gene clusters are present in the genome of *Cupriavidus neocaledonicus* STM 6070, a new species of *Mimosa pudica* microsymbiont isolated from heavy-metal-rich mining site soil

**DOI:** 10.1186/s12864-020-6623-z

**Published:** 2020-03-06

**Authors:** Agnieszka Klonowska, Lionel Moulin, Julie Kaye Ardley, Florence Braun, Margaret Mary Gollagher, Jaco Daniel Zandberg, Dora Vasileva Marinova, Marcel Huntemann, T. B. K. Reddy, Neha Jacob Varghese, Tanja Woyke, Natalia Ivanova, Rekha Seshadri, Nikos Kyrpides, Wayne Gerald Reeve

**Affiliations:** 1 IRD, Cirad, Univ. Montpellier, Interactions Plantes Microorganismes Environnement (IPME), 34394 Montpellier, France; 2 College of Science, Health, Engineering and Education, Murdoch University, Perth, Australia; 3 IRD, UMR LSTM-Laboratoire des Symbioses Tropicales et Méditerranéennes, 34398 Montpellier cedex 5, France; 4 Curtin University Sustainability Policy Institute, Curtin University, Bentley, Australia; 5 DOE Joint Genome Institute, Walnut Creek, USA

**Keywords:** Rhizobia, *Cupriavidus*, Nickel tolerance, HGT, *Mimosa*, Rhizobial biogeography, Heavy metal resistance, Heavy metal efflux

## Abstract

**Background:**

*Cupriavidus* strain STM 6070 was isolated from nickel-rich soil collected near Koniambo massif, New Caledonia, using the invasive legume trap host *Mimosa pudica*. STM 6070 is a heavy metal-tolerant strain that is highly effective at fixing nitrogen with *M. pudica*. Here we have provided an updated taxonomy for STM 6070 and described salient features of the annotated genome, focusing on heavy metal resistance (HMR) loci and heavy metal efflux (HME) systems.

**Results:**

The 6,771,773 bp high-quality-draft genome consists of 107 scaffolds containing 6118 protein-coding genes. ANI values show that STM 6070 is a new species of *Cupriavidus*. The STM 6070 symbiotic region was syntenic with that of the *M. pudica*-nodulating *Cupriavidus taiwanensis* LMG 19424^T^. In contrast to the nickel and zinc sensitivity of *C. taiwanensis* strains, STM 6070 grew at high Ni^2+^ and Zn^2+^ concentrations. The STM 6070 genome contains 55 genes, located in 12 clusters, that encode HMR structural proteins belonging to the RND, MFS, CHR, ARC3, CDF and P-ATPase protein superfamilies. These HMR molecular determinants are putatively involved in arsenic (*ars*), chromium (*chr*), cobalt-zinc-cadmium (*czc*), copper (*cop, cup*), nickel (*nie* and *nre*), and silver and/or copper (*sil*) resistance. Seven of these HMR clusters were common to symbiotic and non-symbiotic *Cupriavidus* species, while four clusters were specific to STM 6070, with three of these being associated with insertion sequences. Within the specific STM 6070 HMR clusters, three novel HME-RND systems (*nieIC cep nieBA*, *czcC2B2A2*, and *hmxB zneAC zneR hmxS*) were identified, which constitute new candidate genes for nickel and zinc resistance.

**Conclusions:**

STM 6070 belongs to a new *Cupriavidus* species, for which we have proposed the name *Cupriavidus neocaledonicus* sp. nov.. STM6070 harbours a pSym with a high degree of gene conservation to the pSyms of *M. pudica*-nodulating *C. taiwanensis* strains, probably as a result of recent horizontal transfer. The presence of specific HMR clusters, associated with transposase genes, suggests that the selection pressure of the New Caledonian ultramafic soils has driven the specific adaptation of STM 6070 to heavy-metal-rich soils via horizontal gene transfer.

## Background

Rhizobia are nitrogen-fixing legume microsymbionts belonging to the alpha and beta subclass of Proteobacteria, and have been named for convenience alpha- and beta-rhizobia [1, 2]. Alpha-rhizobia are common symbionts of most legume species, whereas many of the beta-rhizobial strains have a particular affinity with *Mimosa* hosts [1, 3]. The competitiveness of beta-rhizobial *Paraburkholderia* or *Cupriavidus* strains for nodulation of *Mimosa* spp. varies as a function of the host species and/or ecotypes [4], and of soil characteristics such as nitrogen availability and pH [5, 6].

While *Paraburkholderia* symbionts are considered to be ancient partners of *Mimosa* spp. [7], the *Cupriavidus-Mimosa* symbiosis seems to have evolved more recently [6, 8]. Symbiotic *Cupriavidus* strains belonging mainly to the species *C. taiwanensis* have been isolated from nodules of the invasive species *Mimosa diplotricha* Sauvalle*, Mimosa pigra* L. and *Mimosa pudica* L., with the type strain *C. taiwanensis* LMG 19424^T^ being isolated from a nodule of *M. pudica* growing in Taiwan [6, 9–16]. Strains of *C. necator* and *Cupriavidus* sp. that nodulate the mimosoid legume *Parapiptadenia rigida* and native *Mimosa* spp. in Uruguay and in Texas, USA have also been described [17–19]. *Cupriavidus* strains have so far not been isolated from native species of *Mimosa* growing in Brazil [7] or in India [20], raising questions as to the origins and native hosts of rhizobial *Cupriavidus* species.

Within *Cupriavidus*, several species seem particularly adapted to metal-rich environments [21, 22]. The most well-known and studied strain is *C. metallidurans* CH34^T^, which represents the model bacterium for metal resistance studies [21, 22]. Other *Cupriavidus* species, such as *C. necator* (formerly *C. eutrophus*) H16 [23, 24], are metabolically versatile organisms capable of growth in the absence of organic substrates and able to use H_2_ and CO_2_ as sole sources of energy and carbon [25]. The genome of *C. necator* H16 was shown to display high similarity to the genome of *C. taiwanensis* LMG 19424^T^ [8].

We were interested in questions concerning the origin and adaptation of *M. pudica* microsymbionts found in soils characterized by heavy metal contamination in New Caledonia [13]. *M. pudica*, which originates from the Americas [26], was introduced onto the island probably at the end of the nineteenth century. It has become a serious weed on many Pacific Islands, where it can form dense mats, resulting in land degradation, biodiversity loss and decreased agricultural yield and economic productivity [27, 28]. Conversely, the combination of *M. pudica* and associated *Cupriavidus* rhizobia has been advocated as a novel biosorption system for removing heavy-metal pollutants [29].

A study of rhizobia isolated from New Caledonian *M. pudica* trap hosts identified five different 16S RNA and REP-PCR *Cupriavidus* genotypes (I to V) that nodulated this host [13]. *Cupriavidus* strain STM 6070 is a representative strain of a group of 15 isolates belonging to genotype III. These isolates were obtained from plants grown in a soil characterized by high total nickel concentrations (1.56 g kg^− 1^) that was collected from an active nickel mine site at the bottom of the Koniambo Massif [13]. STM 6070 and the other genotype III isolates, initially ascribed to the *C. taiwanensis* species, are highly nickel-tolerant and appear to be well adapted to the ultramafic soils they were isolated from. Strain STM 6070 was selected as part of the DOE Joint Genome Institute 2010 Genomic Encyclopaedia for Bacteria and Archaea-Root Nodule Bacteria (GEBA-RNB) sequencing project [30, 31], to allow comparative genomic studies concerning the evolution of *Cupriavidus* symbionts and, in particular, their adaptation to metal-rich environments. In this study, whole-genome data of STM 6070 was compared with genomes of symbiotic *Cupriavidus* species [6, 8, 32, 33], non-symbiotic strains of *Cupriavidus* [25, 34–36], and two genomes of the closely related genus *Ralstonia* [37]. Here we show that the STM 6070 genome harbours a multitude of diverse heavy metal resistance (HMR) loci, including putative *ars*, *czc*, *chr*, *cop* and *nre* operons. By comparing the STM 6070 HMR loci to those in other *Cupriavidus* genomes, we identified four gene clusters (clusters B, D, I and J) that are specific to STM 6070 and may be important genetic determinants that contribute to the adaptation of this strain to the heavy-metal-rich ultramafic Koniambo soil in New Caledonia.

## Results and discussion

### General characteristics of *Cupriavidus* strain STM 6070

STM 6070 is a fast-growing, Gram-negative, motile, rod-shaped isolate that forms white-opaque, slightly domed and moderately mucoid colonies within 2–3 days when grown on solid media (Figure S1). Because STM 6070 was trapped from nickel-rich ultramafic soil, we compared its heavy metal tolerance with that of other symbiotic and non-symbiotic *Cupriavidus* strains. The growth of STM 6070 was compared to the growth of *C. metallidurans* CH34^T^ (a model organism for heavy metal resistance [21]) and its heavy metal-sensitive derivative AE104 (CH34^T^ devoid of the plasmids pMOL28 and pMOL30 that confer heavy-metal-resistance [38]) at various concentrations of Ni^2+^ (Figure S2). Of the tested strains, STM 6070 had the highest tolerance to Ni^2+^ and was the only strain capable of growth at 15 mM NiSO_4_.

*C. metallidurans* CH34^T^ grew in the presence of 10 mM NiSO_4_, while AE104 was unable to grow at 3 mM NiSO_4_. Previous studies had established that other symbiotic *C. taiwanensis* strains LMG 19424^T^ from Taiwan [13] and *C. taiwanensis* STM 6018 from French Guiana [6] were also unable to grow at 3 mM NiSO_4_ (data not shown).

In light of the observed Ni^2+^ tolerance of STM 6070, we examined the tolerance of the *Cupriavidus* symbionts to other metal ions. In the presence of Cu^2+^, STM 6070, 6018 and LMG 19424^T^ were able to grow in media containing 1.0 mM Cu^2+^, however, growth of STM 6070 was inhibited from 0.6 mM Cu^2+^ (Figure S3). In addition, STM 6070 was able to grow in media containing 15 mM Zn^2+^, whereas STM 6018 and LMG 19424^T^ were far more sensitive and could not grow at this concentration (data not shown). Since STM 6070 was highly tolerant to Ni^2+^ and Zn^2+^, the genome of this strain was examined, in particular for putative HMR determinants.

### STM 6070 minimum information for the genome sequence (MIGS) and genome properties

The classification, general features and genome sequencing project information for *Cupriavidus* strain STM 6070 are provided in Table S1, in accordance with the minimum information about a genome sequence (MIGS) recommendations [39] published by the Genomic Standards Consortium [40]. The genome sequence consisted of 6,771,773 nucleotides with 67.21% G + C content and 107 scaffolds (Table 1) and contained a total of 6182 genes, of which 6118 were protein encoding and 64 were RNA only encoding genes. The majority of protein encoding genes (81.69%) were assigned a putative function, whilst the remaining genes were annotated as hypothetical. The distribution of genes into COGs functional categories is presented in Table S2.

**Table 1:** Genome Statistics for *Cupriavidus* strain STM 6070

Attribute	Value	% of Total
Genome size (bp)	6,771,773	100.00
DNA coding region (bp)	5,928,188	87.54
DNA G + C content (bp)	4,551,463	67.21
Number of scaffolds	107	
Total gene	6182	100.00
RNA genes	64	1.04
rRNA operons*	1	0.02
Protein-coding genes	6118	98.96
Genes with function prediction	5050	81.69
Genes assigned to COGs	4500	72.79
Genes assigned Pfam domains	5305	85.81
Genes with signal peptides	677	10.95
Genes with transmembrane helices	1402	22.68
CRISPR repeats	1	

*1 copy of 16S rRNA and 4 copies of 5S rRNA

### Phylogenetic placement of STM 6070 within the *Cupriavidus genus*

Previous studies have shown that STM 6070 is most closely related to *C. taiwanensis* LMG 19424^T^ [11] and *C*. *alkaliphilus* ASC-732^T^ [34], according to *recA* phylogenies [13]. This was confirmed by a phylogenetic analysis based on an intragenic fragment of the 16S rRNA gene (Figure S4). To determine the taxonomic placement of STM 6070 at the species level, the whole genome of STM 6070 was compared with sequenced genomes of five non-symbiotic and three symbiotic *Cupriavidus* species (Table S3) to establish the average nucleotide identity (ANI) (Table S4).

ANI [41–43] comparisons showed that the STM 6070 genome displayed the highest ANI values with the *C. taiwanensis* strains STM 6018 and LMG 19424^T^, but the values were lower than the species affiliation cut-off scores (Table S4). This reveals that STM 6070 (and isolates of the same rep-PCR group isolated from New Caledonia soils [13]) represent a new *Cupriavidus* species, for which we propose the name *Cupriavidus neocaledonicus* sp. nov. (i.e. from New Caledonia). The ANI values also suggest that the UYPR2.512 and AMP6 strains represent new *Cupriavidus* species.

### Synteny between genomes

**Fig. 1:**
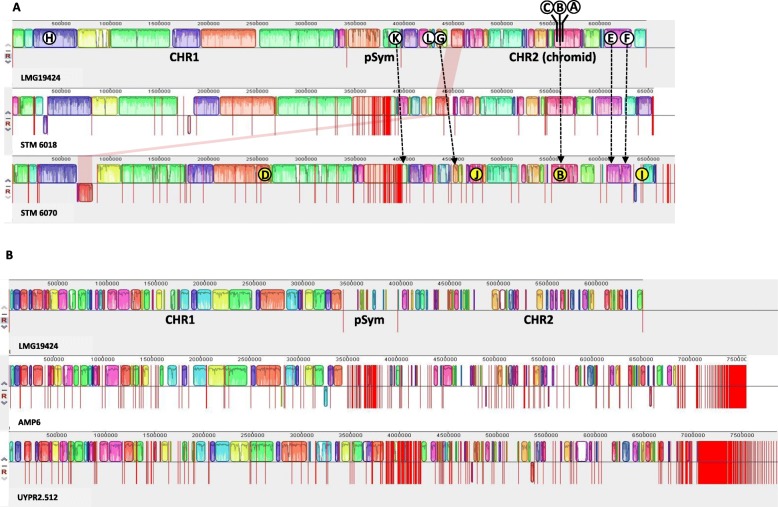
Genome alignments using progressive Mauve software [44]. **a**: scaffolds of the draft genomes of *Cupriavidus neocaledonicus* STM 6070 (STM 6070) and *C. taiwanensis* STM 6018 aligned to the replicons of the finished genome of *Cupriavidus taiwanensis* LMG 19424^T^ (LMG 19424). **b**: scaffolds of the draft genomes of *Cupriavidus* sp. strains AMP6 and UYPR2.512 aligned to the replicons of the finished genome of *Cupriavidus taiwanensis* LMG 19424^T^ (LMG 19424). The blocks in the alignment represent the common local colinear blocks (LCBs) among the compared genomes, and homologous blocks in each genome are shown as identical coloured regions. The vertical red lines represent replicon boundaries for LMG 19424^T^, whereas they represent contig boundaries for the draft genomes. The shaded red region represents a putative genomic rearrangement between CHR2 and CHR1. Circles with numbers represent the location of heavy metal resistance regions identified in this paper found in LMG 19424^T^ (white circles containing letters) and in STM 6070 (yellow circles containing letters). See Fig. 3 for the heavy metal resistance regions. Dashed arrows show the location of the LMG 19424^T^ heavy metal resistance regions in STM 6070

To assess how the observed differences in genome size (6.48–7.86 Mb) affected the distribution of specific genes within the five symbiotic strains of *Cupriavidus*, we used progressive Mauve [44] to align the draft genomes of STM 6070, STM 6018, UYPR2.512 and AMP6 to the finished genome of *C. taiwanensis* LMG 19424^T^ (Fig. 1). The alignments of the STM 6018 and STM 6070 genomes against that of *C. taiwanensis* LMG 19424^T^ showed a high similarity of collinear blocks within the two largest replicons (Fig. 1a), the sequence of the LMG 19424^T^ chromosome 1 (CHR1) being more conserved than that of the chromosome 2 (CHR2 or chromid). We identified eight scaffolds specific to STM 6070 (A3AGDRAFT_scaffold_31.32_C, _43.44_C, _54.55_C, _39.40_C, _104.105_C, _101.102_C, _99.100_C, and _89.90_C) that could not be aligned to the LMG 19424^T^ genome sequence, as well as two STM 6070 scaffolds (A3AGDRAFT_scaffold_84.85_C and _75.76_C) that were absent from LMG 19424^T^ but present in STM 6018. A putative genomic rearrangement was also detected within one scaffold of STM 6070 (A3ADRAFT_scaffold_0.1), in which one part of the scaffold mapped to chromosome CHR1 and another part mapped to the chromid CHR2 of LMG 19424^T^ (see shaded area on Fig. 1a).

In contrast, the genome alignment of UYPR2.512 and AMP6 with LMG 19424^T^ showed important differences in replicon conservation (Fig. 1b). Earlier studies on comparative genomics of *Cupriavidus* species have suggested that the largest CHR1 replicon probably constitutes the ancestral one, while the smaller CHR2 replicon was acquired as a plasmid during the evolution of *Cupriavidus* and gradually evolved to a large-sized replicon following either gene transfer from CHR1 or horizontal gene transfer [35]. Large secondary replicons, or “chromids” [46], such as CHR2, have been detected in many bacterial species and carry plasmid-like partitioning systems [25, 35] and some essential genes, such as rRNA operons and tRNA genes (present in CHR2 of LMG 19424^T^ and the corresponding syntenic region of STM6070). This chromid also carries many genes that are conserved within a genus, and genes conserved among strains within a species. This may well explain the greater degree of sequence divergence observed (Fig. 1) in CHR2 as compared with CHR1 in the symbiotic *Cupriavidus* genomes.

Finally, we observed that whereas most of the LMG 19424^T^ pSym sequence was well conserved in the STM 6018 and STM 6070 genomes (Fig. 1a), only a few LMG 19424^T^ pSym genes (including the *nod*, *nif*, *fix* and *fdx* genes) were conserved across all five genomes. The *M. pudica* microsymbionts (LMG 19424^T^, STM 6018 and STM 6070) had almost identical pSyms (conserved pSym synteny with *nod* genes characterized by 100% protein identity). In contrast, the *Parapiptadenia rigida* (UYPR2.512) and *Mimosa asperata* (AMP6) nodulating strains harboured divergent pSyms (low synteny, with *nod* genes characterized by 80–94 and 95–98.4% protein identity to those of LMG 19424^T^, respectively). Based on phylogenetic analyses of symbiotic and housekeeping loci, our results support the hypothesis that symbiotic *Cupriavidus* populations have arisen via horizontal gene transfer [47].

### Comparisons of *Cupriavidus neocaledonicus* STM 6070 with other sequenced genomes of symbiotic *Cupriavidus*

**Fig. 2:**
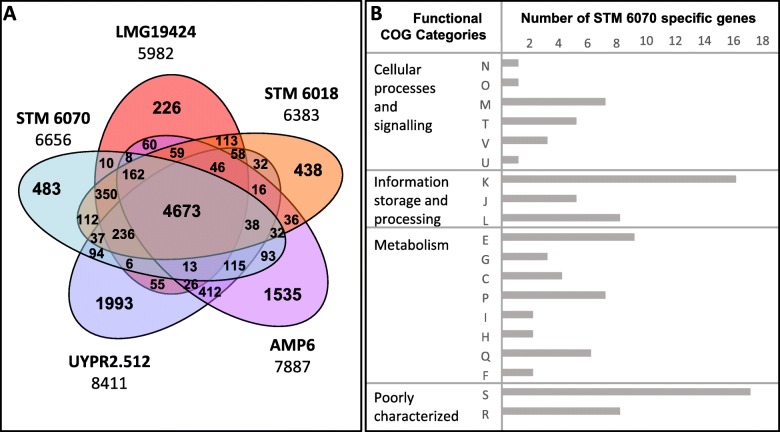
Gene content analysis of the STM 6070 genome. **a**: Venn diagram of gene number counts of symbiotic *Cupriavidus* strains; **b**: functional COG categories of STM 6070 specific genes (107 assigned genes out of 483). STM 6070, *Cupriavidus neocaledonicus* STM 6070; STM 6018, *C. taiwanensis* STM 6018; LMG 19424^T^, *C. taiwanensis* LMG 19424^T^; AMP6, *Cupriavidus* sp. AMP6; UYPR2.512, *Cupriavidus* sp. UYPR2.512. Numbers under the strain names describe the total number of genes for each corresponding genome. The analysis was performed using the “Gene Phyloprofile” tool in the Microscope MaGe platform [48], https://www.genoscope.cns.fr/agc/microscope/mage). The orthologous counterparts in the genomes were detected by applying a minimum of 30% for protein sequences identity over a minimum of 80% of the protein length (> 30% protein MinLrap 0.8)

The comparison of gene orthologues of STM 6070 with those of the symbiotic *Cupriavidus* strains LMG 19424^T^, STM 6018, UYPR2.512 and AMP6, performed using the “Gene Phyloprofile” tool in the Microscope MaGe platform [48] (Fig. 2), showed that these strains have a large core set of 4673 genes, representing from 55.5 to 78.1% of the total number of genes in these organisms (70.2% for STM 6070). Each species harbours a set of unique genes, which range from 226 for LMG 19424^T^ to 1993 for UYPR2.512; larger genomes had a greater number of unique genes (Fig. 2). STM 6070 harbours 483 unique genes, which represent 7.2% of the total number of genes in the genome. The majority of these unique genes (376) encode hypothetical proteins. Only 22.2% of the 483 STM 6070 unique genes could be ascribed to functional COG categories (Fig. 1b). Within the functional COG category “Cellular processes and signaling”, the largest number of genes were found in Cell wall/membrane/envelop biogenesis, Signal Transduction, Defense mechanism and Intracellular trafficking, secretion, and vesicular transport. This may be related to processes required for plant host relationships and bacterial adaptation to the host environment. For example, within functional category M we detected several genes encoding glycosyl transferases, which are putatively involved in biosynthesis of exopolysaccharides and/or polysaccharides, products that have been shown to play a major role in rhizobial infection [49].

Unique STM 6070 genes within the signal transduction category included four genes encoding putative universal stress proteins (UspA family), additional response regulators and a sensor protein (RcsC), while the defense mechanism category includes genes encoding type I and III restriction modification systems, as well as genes encoding multidrug resistance efflux pumps, which could reflect adaptation to ultramafic soils. A high number of specific genes was assigned to “Information storage and processing”. For example, 38 genes encoded putative transcriptional regulators (COG category ‘transcription’) of various families (AraC, CopG, GntR, LacI, LysR, LuxR, MerR, NagC, TetR and XRE), suggesting a requirement for supplementary regulatory mechanisms of cellular and metabolic processes. Finally, a high number of specific genes was assigned to metabolic functions, represented mainly by amino acid, carbohydrate and inorganic ion transport and metabolism, energy production and conversion, lipid metabolism and secondary metabolites biosynthesis, transport and catabolism.

### Metal resistance determinants in the STM 6070 genome

**Fig. 3:**
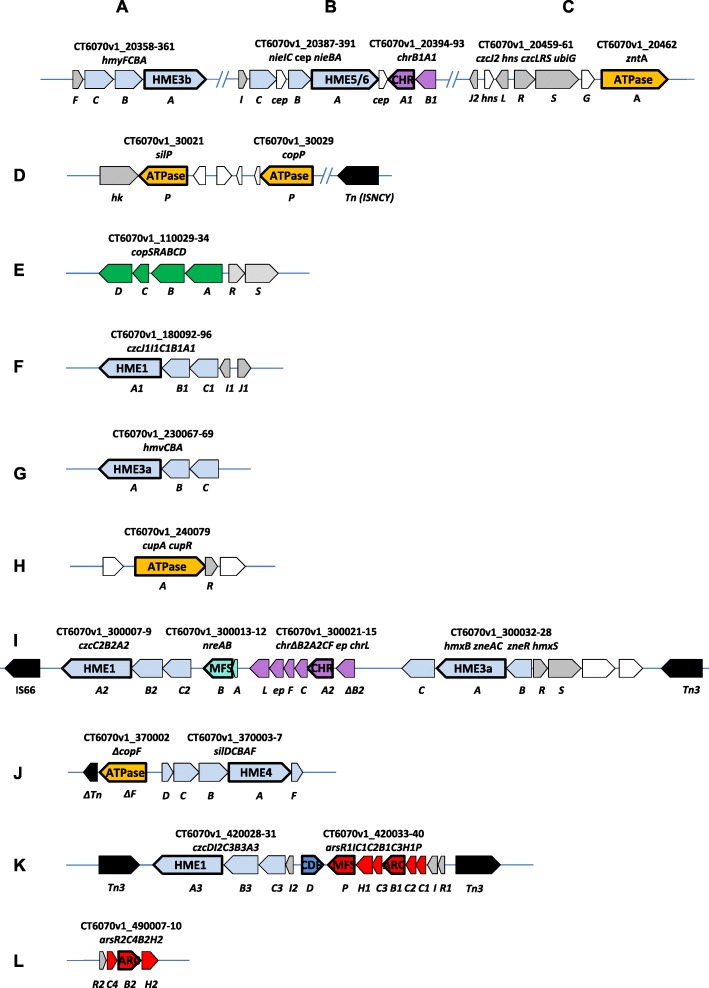
*Cupriavidus neocaledonicus* STM 6070 HMR gene clusters containing annotated putative genes encoding proteins involved in heavy metal efflux (HME). A to L: HMR loci (see also Table S6). Colour coding: light blue, HME-RND systems composed of canonical CBA genes [45]; dark blue, *czcD* encoding a CDF type protein; turquoise, *nre* genes; dark and light grey, putative corresponding regulatory genes; green, *cop* genes; purple, *chr* genes; red, *ars* genes; yellow, P-ATPase encoding genes; white, genes encoding putative proteins of unknown function; black, transposases; *cep*: conserved exported protein; ep, exported protein; hk, histidine kinase. Truncated genes are identified with a delta (Δ) symbol. Thick lines identify genes encoding the transmembrane proteins. Gene coordinates for STM 6070 (CT6070v1_XXXXXX-XX) correspond to the annotation in the MaGe Microscope platform (https://www.genoscope.cns.fr/agc/microscope/mage/viewer.php) (see Table S6 for the corresponding IMG locus tags)

To understand the genetic basis of STM 6070 metal tolerance, we then searched for the presence of common and specific heavy metal resistance (HMR) markers within the genomes of STM 6070 and the other symbiotic *Cupriavidus* species, using the TransAAP tool on the TransportDB website (http://www.membranetransport.org/) [50] to find genes encoding predicted transporter proteins. Given that STM 6070 is nickel- and zinc-tolerant, we were particularly interested in identifying HMR proteins within known transporter superfamilies (Transporter Classification Database: http://www.tcdb.org/) [45, 51, 52]. TransAAP analysis revealed a total of 834 putative transporters within STM 6070, of which 156 were classified within the MFS, CDF, RND, CHR, ACR3 and P-ATPase protein families (Table S5). Of the 156 TransportDB predicted transporters, 23 HME transporter genes were identified in the STM 6070 genome. Based on gene arrangements and homology with characterised HMR loci, a total of 55 structural HMR genes (TransportDB predicted HME genes plus associated genes) were located in 12 clusters (clusters A – L, Fig. 3). The transporter superfamily genes were compared with those described for *C. metallidurans* CH34^T^, *C. necator* H16, and the symbiotic species *C. taiwanensis* LMG 19424^T^ [35], *Cupriavidus* sp. UYPR2.512 and *Cupriavidus* sp. AMP6 (Table 2, Table S6).

**Table 2:** HME determinants in STM 6070 genome and their comparison with those detected in other *Cupriavidus* species

Locus ^**a**^	Proposed gene annotation	IMG Locus tag ^**b**^ A3AGDRAFT_	HM transporter ^**c**^	Putative substrate	Orthologous genes						
					LMG 19424^**T**^	STM 6018	UYPR2.512	AMP6	CH34^**T**^	N-1	H16
A	*hmyFCBA*	00876–00879	HME3b	monovalent cations	+	+	+	+	+	+	+
B	*nieIC cep nieBA*	00906–00910	HME5	nd	+	+	+	+	–	+	+
	*chrB1A1*	00912–00911	CHR	CrO_4_^2−^	+	+	+	–	+	–	–
C	*czcJ2 hns czcLRS ubiG* *zntA*	00978 (zntA)	P-ATPase	Zn^2+^	+	+	+	+	+	+	+
D	*silP*	00999	P-ATPase	Ag	–	–	–	+	–	–	+
	*copP*	01005	P-ATPase	Cu^2+^	–	–	+	+	+	–	+
E	*copSRABCD*	02911–02906	–	nd	+	+	+	+	+	+	+
F	*czcJ1I1C1B1A1*	03877–03882	HME1	Co^2+^, Zn^2+^, Cd^2+^	+	+	+	+	+	+	+
G	*hmvCBA*	04395–04397	HME3a	divalent cations	+	+	+	+	+	+	+
H	*cupA* *cupR*	04504	P-ATPase	Cu^2+^	+	+	+	+	+	+	+
I	*czcC2B2A2*	04975–04973	HME1	nd	+	+	+	+	+	+	+
	*nreAB*	04978–04977	MFS	Ni^2+^	–	–	+	+	+	–	–
	*ΔchrB 2A2CF exp chrL*	04985–04980	CHR	CrO_4_^2−^	+	+	+	+	+	+	+
	*hmxB zneAC zneR hmxS*	04993–04989	HME3a	nd	+	+	+	+	+	+	+
J	*ΔcopF*	05405	P-ATPase	Cu^2+^	+	+	+	+	+	+	+
	*silDCBAF*	05406–05409	HME4	Cu^2+^, Ag	+	+	+	+	+	+	+
K	*czcDI2C3B3A3*	05655–05651	CDF + HME1	Co^2+^, Zn^2+^, Cd^2+^	+	+	+	+	+	+	+
	*arsR1IC1C2B1C3H1P*	05663–05656	ARC3	nd	+	+	–	+	+	+	+
			+MFS		+	+	–	+	+	+	+
L	*arsR2C4B2H2*	05853–05856	ARC3	nd	+	+	–	+	+	+	+
Not in HMR clusters	*dmeF*	04892	CDF + HME1	nd	+	+	–	+	+	+	+
	*fieF1*	03199	CDF + HME1	nd	+	+	–	+	+	+	+
	*fieF2*	06164	CDF + HME1	nd	+	+	–	+	+	+	+

Footnote: STM 6070, *Cupriavidus* sp. STM 6070; LMG 19424T, *C. taiwanensis* LMG 19424T; STM 6018, *C. taiwanensis* STM 6018; UYPR2.512, *Cupriavidus* sp. UYPR2.512; AMP6, *Cupriavidus* sp. AMP6; CH34T, *C. metallidurans* CH34T; N-1, *C. necator* N-1 T

^a^, HM loci names from Fig. 2 and Table S6

^b^, IMG Locus tag (JGI);

^c^, classification of the annotated HM proteins from Table S5

#### Major facilitator superfamily (MFS) proteins

The MFS is one of the two largest families of membrane transporters found in living organisms. Within the MFS permeases, 29 distinct families have been described, each transporting a single class of compounds [53]. Of the 106 STM 6070 TransAAP-identified genes encoding putative MFS proteins, two genes (*nreB* and *arsP*) were associated with HME functions. The *nreB* gene located in the *nreAB* operon (cluster I), and the *arsP* gene located in the *arsRIC1C2BC3H1P* operon (cluster K), encode putative nickel and arsenic efflux systems, respectively (Fig. 3) [45].

#### Cation diffusion facilitator (CDF) proteins

The CDF proteins are single-subunit systems located in the cytoplasmic membrane that act as chemiosmotic ion-proton exchangers [52]. They include HMR proteins such as CzcD, which provides resistance to cobalt, zinc and cadmium [45]. Four genes encoding CDF proteins were detected in the STM 6070 genome (Table S6), but only one, *czcD*, is located in an HME cluster (*czcDI2C3B3A3,* cluster K) (Fig. 3). This locus encodes a CDF efflux protein with 67.2% identity to CH34^T^ CzcD, which mediates the efflux of Co^+ 2^, Zn^+ 2^, and Cd^+ 2^ ions [54]. The second CDF gene (*dmeF*) encodes an efflux protein with highest identity (76.1%) to the CH34^T^ DmeF protein, which has a role in cobalt homeostasis and resistance [54], while the other two CDF genes (*fieF1* and *fieF2*) encode efflux proteins with homology to CH34^T^ FieF (70.8 and 69.8% identity, respectively). FieF has a role in ferrous iron detoxification but was also shown to mediate low level resistance to other divalent metal cations such as Zn^2+^ and Cd^2+^ [55, 56].

#### Resistance-nodulation-cell division (RND)-HME systems

The RND-HME transporters are transmembrane proteins that form a tripartite protein complex consisting of the RND transmembrane transporter protein (component A), a membrane fusion protein (MFP) (component B), and an outer membrane factor (OMF) protein (component C). These components export toxic heavy metals from the cytoplasm, or the periplasm, to the outside of the cell and have been designated as CBA efflux systems, or CBA transporters [45], to differentiate them from ABC transporters. Within a CBA system, the RND transmembrane and MFP proteins [45, 57], mediate the active part of the transport process, determine the substrate specificity, and are involved in the assembly of the RND-HME protein complex.

The RND-HME transmembrane proteins contain a large periplasmic loop flanked by 12 transmembrane α-helices, TMH I to TMH XII [45]. They are classified into different groups according to the signature consensus sequence located in TMH IV, which is essential for proton/cation antiport and is used to predict the heavy metal substrate specificity [45, 58]. The five classes of efflux systems and their predicted heavy metal substrates include: HME1 (Co^2+^, Zn^2+^, Cd^2+^), HME2 (Co^2+^, Ni^2+^), HME3a (divalent cations), HME3b (monovalent cations) HME4 (Cu^+^ or Ag^+^) and HME5 (Ni^2+^) types [45, 59, 60].

Our phylogenetic analysis of the eight TransAAP predicted STM 6070 RND proteins, together with the analysis of the conserved motifs within the proteins, suggests that three of these proteins belong to the HME1 class, two belong to the HME3a class and the remaining three proteins belong to the HME3b, HME4 and HME5 classes, respectively (Fig. 3 and Table S6). The STM 6070 genome lacks genes encoding the HME2-type transmembrane proteins, such as the *C. metallidurans* CH34^T^ CnrA and NccA, which are involved in heavy metal resistance and have predicted substrate specificity for cobalt and nickel [45].

#### RND-HME1

**Fig. 4:**
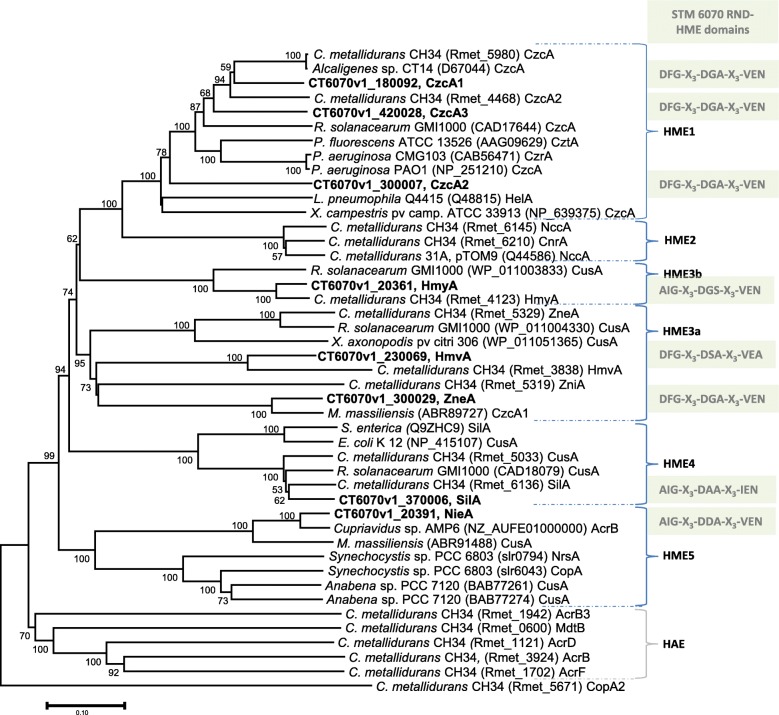
Phylogenetic tree of the RND transmembrane proteins (component A of the CBA transport system) from *Cupriavidus neocaledonicus* STM 6070 (in bold) and from sequenced genomes of reference *Cupriavidus* (*C.*), *Escherichia* (*E*.), *Legionella* (*L.*), *Microbacterium* (*M*.), *Pseudomonas* (*P*.), *Ralstonia* (*R*.) and *Xanthomonas* (*X*.) and other named reference strains. The HME class of the protein is designated according to the current classification scheme [45, 59, 60]. HME1 to 5 represent five classes of HME systems, HAE represents here an RND protein group involved in in export of hydrophobic and amphiphilic compounds. The evolutionary history was inferred by the Neighbor-Joining method with a bootstrap consensus tree inferred from 500 replicates. The evolutionary distances were computed using the Poisson correction method and are presented as the number of amino acid substitutions per site. The rate variation among sites was modelled with a gamma distribution (shape parameter = 1). Evolutionary analyses were conducted in MEGA6. STM 6070 HME locus tags are displayed in Fig. 2 and Table S6. GenBank accession numbers or locus tags are given in brackets. For *C. metallidurans* CH34^T^ only the gene numbers of the annotated genome (NC_007973) are given

STM 6070 contained 3 RND-HME1 encoded proteins (CzcA1-A3), characterized on the basis of homology to the canonical CzcA proteins of CH34^T^. STM 6070 CzcA1 and CzcA3 grouped with CH34^T^ CzcA and CczA2, while STM 6070 CzcA2 formed an outgroup (Fig. 4). The *C. necator czcA1* gene was within an operon located in cluster F (Fig. 3) and annotated as *czcJ1I1C1B1A1*. In addition to the *czcCBA* genes, this cluster contained a *czcI1* homolog to a transcriptional regulator that has been shown to control the expression of *czcC1B1A1* [45, 61] and a *czcJ1* homolog, which was reported to be strongly induced by Cd^2+^, Cu^2+^, Ni^2+^, and Zn^2+^ [35, 62]. This operon was located in a genomic region showing high synteny with corresponding regions in the other symbiotic *Cupriavidus* strains and in *C. necator* N-1, and the STM 6070 CzcA1 protein showed high identity with other *Cupriavidus* CzcA orthologues (Table S6). In *C. metallidurans* CH34^T^, the corresponding *czc* cluster (*czcMNICBADRSEJ*, locus tags Rmet_5985–74) is located on the plasmid pMOL30 and contains additional genes that are not found in STM 6070 [35]. The second STM 6070 RND-HME1 efflux system (*czcC2B2A2*) formed part of a large group of HMR loci within cluster I (Fig. 3). Immediately upstream of *czcC2B2A2* is a *nreB* gene, encoding a putative nickel resistance MFS protein. A similar arrangement has been observed for the CH34^T^ *nccCBA nreB* cluster found on plasmid pMOL30 [35]. Cluster I was delimited by transposases and no conserved syntenic arrangement with the six other *Cupriavidus* genomes was observed (Table 2). The third STM 6070 RND-HME1 efflux system (*czcD czcI2C3B3A*) was located within cluster K (Fig. 3). The *czcI2* and *czcD* components encode putative regulator and CDF proteins, respectively. This cluster, which was also delimited by two Tn*3* transposases, had conserved synteny to corresponding regions in the genomes of *Cupriavidus* spp. LMG 19424^T^ and STM 6018, but not to AMP6 and UYPR2.512.

#### RND-HME3a

STM 6070 contained two putative RND-HME3a efflux systems located in clusters G and I. Cluster G contained an *hmv* operon, located in a region that was syntenic to corresponding regions in symbiotic *Cupriavidus* and *C. necator* N-1. Although the region was not syntenic in CH34^T^, the encoded HmvCBA proteins all had high identity with STM 6070 HmvCBA proteins [35, 63], however, the role of the CH34^T^ proteins in heavy metal efflux has yet to be determined [35].

Cluster I contained a putative zinc efflux RND-HME3a system, annotated as *hmxB zneAC*, with the associated upstream genes *zneRhmxS* encoding a two-component sensor regulatory system. The encoded proteins had low identity (38–44%) to corresponding proteins in other *Cupriavidus* genomes, however, although the BAC gene arrangement is atypical to the characterised RND-HME CBA transporter gene arrangement, it is the same as that described in the characterised CH34^T^ HME3a zinc efflux system *zneSRBAC* [35, 57, 64] (Table S6). The STM 6070 ZneA protein contained highly conserved amino acids identified in the active and proximal heavy metal-binding sites of the characterised CH34^T^ ZneA protein [64] (Table S7). Based on conservation of the essential amino acid residues, these proteins would be divalent cation transporters, putatively involved in zinc efflux. Interestingly, the highest similarity to the STM 6070 HmxB ZneAC proteins (70, 86.5 and 69.5%, respectively) was to encoded proteins of the marine betaproteobacterium *Minibacterium massiliensis*, within an operon of similar architecture but of unknown function and substrate specificity [65].

#### RND-HME3b

An RND-HME3b *hmyFCBA* efflux system was identified in cluster A (Fig. 3). This operon showed high identity to a corresponding CH34^T^ *hmyFCBA* cluster (locus tags Rmet_4119–4123), located on the chromid, and was also highly conserved in the four symbiotic *Cupriavidus* strains and *C. necator* N-1. The role of the Hmy efflux system in *Cupriavidus* is currently unknown and this system is likely to be inactive in CH34^T^ since *hmyA* in this strain is insertionally inactivated by IS*1088* [66]. However, in the characterized *Escherichia coli* metal cation-transporting efflux system CusCFBA, *cusF* encodes a small auxiliary protein that is required for full resistance to copper and silver [67]. A similar role is predicted for the *Cupriavidus hmyF* even though there is low identity (< 30%) to *E. coli cusF*.

#### RND-HME4

An RND-HME4 *silDCBAF* efflux system was identified in cluster J and has been suggested to be important for monovalent cation efflux in CH34^T^ [68]. No syntenic regions were identified in the other *Cupriavidus* genomes. However, this operon is similar to the CH34^T^ pMOL30 *silDCBA* operon (Rmet_5030–5034), which encodes a putative silver efflux system, and to the CH34^T^ chromid *cusDCBAF* operon (Rmet_6133–6136), which encodes a putative copper efflux system [68]. Similar operons were also identified in the STM 6018, AMP6, N-1 and H16 genomes.

#### RND-HME5

An RND-HME5 *nieIC cep nieBA* efflux system, identified in cluster B, was located 28 kb downstream of cluster A. This operon included a gene encoding a conserved exported protein (*cep*) situated between the *nieC* and *nieB* structural genes, disrupting the typical RND CBA operon arrangement. Among the *Cupriavidus* strains, a similar operon structure was found only in the AMP6 genome, with the structural proteins displaying high identity to the corresponding STM 6070 proteins. This operon structure was also found in the genome of *M. massiliensis* [65], with the encoded proteins having 41 to 79% protein identity with those of STM6070. As there are no RND-HME5 efflux systems present in CH34^T^, the protein encoded by STM 6070 *nieA* was compared with the characterized RND-HME5 proteins NrsA (involved in nickel resistance) and CopA (involved in copper resistance) of the cyanobacterium *Synechocystis sp*. PCC 6803 [69, 70]. The phylogenetic analysis (Fig. 4) shows that although these proteins possess a common ancestor, they form two well separated clades, one comprising the HME5 proteins of STM 6070, AMP6 and *M. massiliensis*, and the second containing the NrsA and CopA of PCC 6803 together with RND-HME5 proteins from the cyanobacterium *Anabena sp*. PCC 7120 [71]. The betaproteobacterial and cyanobacterial RND-HME5 proteins share less than 41% identity, resulting in totally different amino acids involved in putative proximal and distal metal-binding sites, as well as differences in the consensus sequence of the TMHIV α-helice (Table S7). Of particular interest was the finding that the three histidines, which are present in the proximal site of NieA and in the proteins of this clade, form part of conserved HAEGVH and HRLDH motifs, and match with putative nickel-binding motifs H-X4-H and H-X3-H that are predominant in Ni-binding proteins, as described for the Ni-binding proteins of *Streptococcus pneumoniae* [72]. Based on these findings, we suggest that this *nieIC cep nieBA* operon encodes a new RND-HME system (class 6) putatively involved in nickel efflux and represents an interesting candidate for knockout mutation to determine if it is a major determinant of nickel tolerance in STM 6070.

#### Chromate ion transporter (CHR) proteins

The CHR proteins efflux chromate from the cytoplasm through an indirect active transport process [73]. Two STM 6070 genes (*chrA1* and *chrA2*) were identified as encoding putative CHR proteins. The STM 6070 ChrA1 and ChrA2 proteins showed higher identity to each other than to the CH34^T^ pMOL28 and chromid ChrA proteins. The *chrB1A1* operon (cluster B) was located up-stream of the putative RND-HME5 efflux system *nieIC-cep-nieBA* (Fig. 3). This *chr* operon was conserved in the genomes of the symbiotic *Cupriavidus* strains LMG 19424^T^ and STM 6018, forming part of a large synteny block. The second *chr* operon, annotated as *chrB2A2CF-cep-chrL (chrY)*, was located in cluster I, along with the RND-HME efflux systems *czcC2B2A2* and *hmxB-zneAC* and the *nreAB* operon (Fig. 3). In addition to *chrB2A2*, this operon contained four other genes: *chrC*, encoding a putative superoxide dismutase that may reduce chromate and thereby decrease chromate toxicity [74]; *chrF*, encoding a putative transcriptional repressor [74]; *cep*, encoding a conserved exported protein containing a Concanavalin A-like lectins/glucanases domain; and finally, *chrL*, encoding a lipoprotein (protein family, LppY/LpqO [75]) with 71.1% identity to CH34^T^ ChrL (also annotated as CH34^T^ ChrY). Corresponding gene clusters were identified in the UYPR2.512 and CH34^T^ genomes. In CH34^T^ the corresponding *chrL* (*chrY*) gene (locus tag Rmet_6195) is induced by chromate [35]). Deletion of *chrL* in the Gram-positive *Arthrobacter* sp. strain FB24 resulted in a noticeable decrease in chromate resistance [75]. The STM 6070 *chr* operon lacks the *chrE*, *chrO*, *chrN*, *chrP* and *chrZ* orthologues found in the corresponding CH34^T^ *chr* operon. The different chromate resistance genes might affect tolerance to chromate, or to another metal-oxyanion [35]. The STM 6070 *chrB2* gene appears to be inactivated by an insertion that changes the reading frame after 214 amino acids, and shortens the protein to only 293 amino acids, instead of the full length 324 amino acid protein encoded by CH34^T^ *chrB*. Since ChrB seems to be important for chromate resistance in CH34^T^ [76], the tolerance of STM 6070 to chromate might be compromised. Indeed, in our experimental conditions STM 6070 only showed slight tolerance to Cr^6+^ (0.1 mM) [13].

#### Arsenical Resistance-3 (ACR3) proteins

The ACR3 family includes permeases involved in arsenate resistance. The two STM 6070 ACR-3 type *arsB1* and *arsB2* genes are located in two *ars* operons encoding putative arsenate detoxification systems. The first operon is located down-stream of the *czc* operon in cluster K. Genes in this *ars* operon had high identity with genes of the CH34^T^ *arsMRIC2BC1HP* operon encoding an arsenite and arsenate detoxifying system [62, 77]. This *ars* cluster encoded a putative arsenite/arsenate transcriptional regulator/repressor (ArsR), a glyoxalase family of proteins (ArsI), three arsenate reductases (ArsC1, ArsC2, ArsC3), an arsenite efflux pump belonging to the ACR3 class of permeases (ArsB1), a NADPH-dependent FMN reductase (ArsH1), and a putative permease from the major facilitator family (MFS) (ArsP) [77]. The operon was highly conserved in the *Cupriavidus* symbionts LMG 19424^T^ and STM 6018 and formed a large syntenic region. The second *ars* operon *arsR2C4B2H2*, in cluster L, was present in all other *Cupriavidus* genomes except UYPR2.512, but was missing several genes (*arsI*, *arsC* and *arsP*) found in the cluster K operon.

#### P-type ATPase proteins

P-type ATPases directly utilise ATP to export metal ions from the cell cytoplasm. Of the ten STM 6070 genes assigned to the P-type ATPase protein family (Table S5), five encoded P-type ATPases putatively involved in HME (Fig. 2 and Table 2). The *copF* P-type ATPase gene in cluster J was located upstream of the *silDCBAF* operon and could encode an essential copper efflux component, as shown for CH34^T^ [38]. However, the STM 6070 CopF appears to be truncated in its C-terminus and thus may not be functional. Two P-type ATPase-encoding genes were identified in cluster D and annotated as *silP* and *copP*. The encoded proteins had very low identity with proteins of the *Cupriavidus* genomes (Table S6), except for one P-type ATPase protein from AMP6 with 86% identity with the CopP protein. The proteins had higher identity with P-type ATPases encoded by *C. necator* H16, annotated as SilP (86%) and CopP (94.7%), and putatively involved in silver and copper ion transport, respectively [25]. Within cluster H, a P-type ATPase-encoding gene, annotated as *cupA*, was located next to a regulatory gene, *cupR* (Fig. 3), in a conserved large syntenic block common to all compared *Cupriavidus* strains, with high identity between corresponding genes. The *cupA* and *cupR* genes are putatively involved in copper ion transport. Finally, *zntA* was located within cluster C in a group of genes annotated as *czcJ2-hns-czcLRS-ubiGI-zntA*. Genes in this cluster had high identity with loci in two gene clusters in CH34^T^ that have been annotated as *zntA czcICΔB* (locus tags Rmet_4594–4597) and *czcBA ubiG czcSRL* IS *hns mmmQ* (locus tags Rmet_4469–4461), respectively. These CH34^T^ clusters encode an RND system (*czcICBA*), the ZntA ATPase, a two-component regulatory system CzcRS and a 3-demethylubiquinone-9 3-methyltransferase (UbiG) [35, 63]. UbiG participates in the biosynthesis of ubiquinone and its activity could be related to the sensor kinase activity of the two-component system CzcRS [78, 79]. The *czcL*, *hns* and *mmmQ* genes encode an unknown protein, an H-NS like protein and a small stress responsive protein, respectively. Genes in the second CH34^T^ cluster may be inactivated by an insertion sequence located between *czcL* and *hns*. The synteny of the STM 6070 cluster C is perfectly conserved in the genomes of the four symbiotic *Cupriavidus* strains, suggesting that it is functional, but it is devoid of the *czcCBA* RND system found in the corresponding CH34^T^ cluster. The role of the regulatory loci *czcLRS-ubiGI*, with regard to *zntA* expression, would thus be interesting to determine.

#### Other mechanisms of cation detoxification (not included in TransAAP)

The search for further heavy metal resistance determinants in STM 6070 that were orthologous to those described in CH34^T^ led to identification of a copper-resistance operon *copRSABCD* (cluster E). This had a similar structure to the CH34^T^ *cop* cluster (*copS2R2A2B2C2D2*) located on the chromid, which encodes a copper-resistance mechanism that is thought to sequester copper outside the cytoplasm [80, 81]. CopSR is a two-component sensor-regulator system and CopA is a putative multi-copper oxidase thought to oxidize Cu^1+^ to Cu^2+^. CopA proteins contain several motif variants of MGGM/MAGM/MGAM/MSGM, possibly involved in binding numerous Cu^1+^ ions, as determined for *Pseudomonas syringae* CopA [82]. CopA is exported to the periplasm by the twin-arginine translocation pathway [81], where it may interact with an outer-membrane protein CopB, providing the minimum system required for low level copper resistance. CopD is a membrane protein involved in transfer of Cu^1+^ from the periplasm to the cytoplasm for CopA binding [80, 81]), and CopC is thought to regulate copper uptake by CopD. The STM 6070 CopA protein shows 75.8% identity to both CH34^T^ CopA1 (pMOL30) and CopA2 (chromid) proteins. Interestingly, the alignment of corresponding proteins reveals the presence of a histidine-rich sequence (GHG GHS GDS GHS GDS (GHS)_5_ GDS GHG AHA GHG) located in the middle of the methionine-rich CopA motif in the STM 6070 protein, which is absent from other CopA sequences deposited in the NCBI database. The *Escherichia coli* HRA-1 and 2, *Enterococcus hirae* CopB [83] and *Rhizobium leguminosarum* ActP [84] Cu-exporting P-type ATPase proteins also contain histidine-rich leaders, which we postulate bind to copper ions. The STM 6070 CopRSABCD putative copper sequestration system may provide a second line of defence against copper toxicity and is particularly well conserved in all of the symbiotic *Cupriavidus* isolates.

#### Location of HMR determinants

The detected STM 6070 HMR determinants in the 12 clusters (A to L, Fig. 3, Table 2) were assigned to putative replicons of the STM 6070 genome, following alignment of contigs to the finished LMG 19424^T^ genome. Two clusters (D and H) could be assigned to chromosome 1 (CHR1), one cluster (K) to the pSym, and nine clusters (A, B, C, E, F, G, I, J and L) to CHR2 (chromid, Figure S5). Therefore, STM 6070 appears to carry the great majority of its HMR clusters on CHR2. In contrast *C. metallidurans* CH34^T^ harbours 8 out of 24 HMR clusters on CHR2 (chromid) [35, 45, 63]. The genome synteny comparison revealed that six of the STM 6070 HMR clusters (A, C, E, F, G and H) are common to symbiotic and non-symbiotic *Cupriavidus* genomes. STM 6070 HME gene products from clusters A, C, E, F, G and H displayed highest identity (93 to 100%) with corresponding proteins of *C. taiwanensis* isolates (LMG 19424^T^ and STM 6018, Table S6), reflecting the taxonomic relationship with *C. taiwanensis*.

Synteny analysis indicated that the specific STM 6070 HMR clusters B, D, I and J were absent from all other analysed *Cupriavidus* genomes, although some of the HMR genes within these clusters had orthologues (35 to 89% of encoded protein identity) in the genomes of the other *Cupriavidus* strains. Cluster K was perfectly conserved within the LMG 19424^T^ and STM 6018 genomes (100%) in a large syntenic region, whereas it was absent from the AMP6 and UYPR2.512 genomes. Only the separate *czc* and *ars* operons from cluster K were detected in the non-symbiotic *Cupriavidus* genomes, with encoded protein identities of 76–77% and 83–88%, respectively, to the STM 6070 *czc* and *ars* operon encoded proteins. This observation can be explained by the location of cluster K on the pSym, which, as proposed recently [47], seems to be largely shared between *M. pudica* microsymbionts of different genomic backgrounds. Indeed, we demonstrated by the progressive Mauve alignment (Fig. 1) that the pSym seems to be conserved in the genomes of the *M. pudica*-nodulating LMG 19424^T^, STM 6018 and STM 6070, in contrast to genomes of AMP6 and UYPR2.512, which nodulate different mimosoid legumes and harbour totally different symbiotic plasmids.

The analysis of genes adjacent to HMR clusters revealed that for clusters D and J contained a transposase-encoding gene at one end of the cluster and clusters I and K were flanked by transposase-encoding genes (Fig. 3). Analysis of the GC% using a two-tailed Mann-Whitney U test revealed that cluster D and J did not contain a significantly different GC% (*P*-value > 0.01) in comparison to the average GC% of the genome. In comparison, clusters I and K did contain a significantly different GC% (P-value < 0.01) in comparison to the average GC% of the genome. This suggests acquisition of the clusters by horizontal gene transfer (HGT) for clusters I and K. Cluster I, located on the chromid, is the largest of these clusters (of approximately 25 kb), flanked by transposases of the Tn*3* and IS66 type, and carries four different HMR determinants, including *czcC2B2A2* and *hmxB zneAC*. Cluster K is flanked by two Tn*3* transposases, however, unlike Cluster I there is a high conservation of architecture and gene identity with the closely related *C. taiwanensis* strains (LMG 19424^T^ and STM 6018). This may indicate that Cluster I contains HME determinants that are important for survival in the New Caledonian ultramafic soils. In *C. metallidurans*, the acquisition of mobile genetic elements that contain metal resistance genes appears to be a strategy important for its adaptation to environments that contain elevated levels of heavy metals [62, 85].

In contrast, no transposases or insertion sequences could be found around cluster B, or more particularly, around the operon *nieIC cep nieBA*). This operon, which is absent from LMG 19424^T^ and STM 6018 genomes, is located in a large highly conserved region, suggesting a gene loss from *C. taiwanensis* genomes. Interestingly, *nieIC cep nieBA* (cluster B) and *hmxB zneAC* (cluster I), two unique RND-HME systems in terms of operon structure and protein sequences, showed significant structure and protein sequence similarity with two operons from the genome of *M. massiliensis* [65].

## Conclusion

New Caledonian *Cupriavidus* microsymbionts isolated from *Mimosa pudica* nodules belong to one of five REP-PCR genotypes, which all harbour identical symbiotic *nodA* and *nifH* genes [13] but display different metal tolerance phenotypes. Fifteen strains belonging to the REP-PCR genotype III were found to be the most nickel-tolerant. The current study presents an analysis of the genome of strain STM 6070, a representative of the REP-PCR genotype III. STM 6070 was originally placed within *C. taiwanensis* on the basis of 16S and *recA* phylogenies [13], however, our analysis, combined with the genetic and phenotypic data described by Klonowska and colleagues [13], has revealed that STM 6070 represents a new species of *Cupriavidus*, for which we propose the name *Cupriavidus neocaledonicus* sp. nov.

The major aim of this study was to gain insights into the molecular basis of the tolerance of *C. neocaledonicus* to high levels of nickel and zinc. The genome of *C. neocaledonicus* STM 6070 contains a very large number of diverse putative HMR determinants belonging to the RND, MFS, CHR, ARC3, CDF and P-ATPase protein superfamilies (Table 2). These constitute putative efflux systems or ion pumps involved in arsenic (2 *ars* operons), chromium (2 *chr* operons), cobalt-zinc-cadmium (2 *czc* operons), copper and/or silver (*copA*, *copP*, and *silA* genes), and nickel (1 *nre* operon and 1 *nie* operon) tolerance. The HMR determinants are clustered in 12 loci (cluster A to cluster L) of which two clusters seem to be localised in CHR1, nine on CHR2 (chromid) and one on the pSym (Figure S5).

Among these clusters, six (A, C, E, F, G and H) are common to both symbiotic and non-symbiotic genomes, with the different levels of sequence similarity suggesting their presence in a bacterial ancestor and possible evolution under different evolutionary pressures. Conversely, cluster K, on the pSym, was present only in STM 6070 and the *C. taiwanensis* strains. The 100% identity of cluster K encoded proteins among the STM 6070, LMG 19424^T^ and STM 6018 genomes could be explained by the “recent” transfer of pSym between the *M. pudica* microsymbionts, in accordance with the findings of Parker [47].

Four of the HMR clusters (B, D, I, and J) are specific to the STM 6070 genome and we propose that these clusters contain genes that are determinants for the adaptation of *C. neocaledonicus* to high concentrations of nickel and zinc in Koniambo soil in New Caledonia. Indeed, within clusters B and I, the identified *nie*, *czc2* and *zne* operons (encoding RND-HME5, −HME1 and -HME3a, efflux systems respectively) constitute good candidates for nickel and zinc tolerance molecular determinants. Moreover, the finding that at least four HMR clusters (D, I, J and K) are directly associated with insertion elements suggests that mobile genetic elements play an important role in adaptation of the STM 6070 genome to the New Caledonian environment. Insertion elements have previously been found to play a role in enabling the host to adapt to new environmental challenges, and to contribute to the genetic adaptation of *C. metallidurans* to toxic zinc concentrations [85, 86]. Future work involving a targeted mutagenesis study should allow us to determine the precise role of the newly identified HMR operons in STM 6070 and will provide an understanding of the specific molecular determinants required for the evolution and adaptation of these bacterial symbionts to the heavy-metal-rich New Caledonian soils.

## Methods

### Bacterial strains and growth conditions

All strains used in this study can be found in Table S3. *C. neocaledonicus* STM 6070 was isolated using *M. pudica* as a trap-host, as previously described, from a soil characterized by high total nickel concentrations (1.56 g kg^− 1^) collected at the bottom of the Koniambo Massif, where active nickel mines are located [13]. Bacterial isolates were sub-cultured on yeast mannitol agar plates (YMA, Vincent, 1970) and incubated at 28 °C for 48 h. For long-term maintenance, bacterial strains were grown in YM broth and preserved in 20% glycerol at − 80 °C. For the comparison of metal tolerance, bacteria were grown in 30 mL liquid 284 Tris-culture medium [19] amended with NiSO_4_ (0, 3, 5, 10 and 15 mM)_,_ Cu (NO_3_)_2_ (0, 0.3, 0.6 and 1.0 mM) and ZnSO_4_ (0, 3, 5, 10 and 15 mM) at 28 °C on a gyratory shaker set to 150 rpm. Bacterial growth was monitored by measuring the OD_600nm_ in a spectrophotometer.

### Genomic DNA preparation

*C. neocaledonicus* STM 6070 was streaked onto TY solid medium [87] and grown at 28 °C for 3 days to obtain well grown, well separated colonies, then a single colony was selected and used to inoculate 5 ml TY broth medium. The culture was grown for 48 h on a gyratory shaker (200 rpm) at 28 °C. Subsequently, 1 ml was used to inoculate 60 ml TY broth medium that was incubated on a gyratory shaker (200 rpm) at 28 °C until an OD_600nm_ of 0.6 was reached. DNA was isolated from 60 ml of cells using a CTAB bacterial genomic DNA isolation method [87]. Final concentration of the DNA was 0.5 mg ml^− 1^.

### Genome sequencing and assembly

The genome of *C. taiwanensis* STM 6070 was sequenced at the Joint Genome Institute (JGI) using Illumina technology [88]. An Illumina standard shotgun library was constructed and sequenced using the Illumina HiSeq 2000 platform which generated 26,402,396 reads totaling 3960.4 Mbp. All raw Illumina sequence data was passed through DUK, a filtering program developed at JGI, which removes known Illumina sequencing and library preparation artifacts (Mingkun, L., Copeland, A. and Han, J., unpublished). The following steps were then performed for assembly: (1) filtered Illumina reads were assembled using Velvet [89] (version 1.1.04), (2) 1–3 kb simulated paired end reads were created from Velvet contigs using wgsim (https://github.com/lh3/wgsim), (3) Illumina reads were assembled with simulated read pairs using Allpaths–LG [90] (version r39750). Parameters for assembly steps were: 1) Velvet (−-v --s 51 --e 71 --i 4 --t 1 --f “-shortPaired -fastq $FASTQ” --o “-ins_length 250 -min_contig_lgth 500”) 10) 2) wgsim (−e 0–1100–2100 -r 0 -R 0 -X 0) 0) 3) Allpaths–LG (PrepareAllpathsInputs:PHRED64 = 1 PLOIDY = 1 FRAGCOVERAGE = 125 JUMPCOVERAGE = 25 LONGJUMPCOV = 50, RunAllpath-sLG: THREADS = 8 RUN = stdshredpairs TARGETS = standard VAPIWARNONLY = True OVERWRITE = True). The final draft assembly contained 107 scaffolds. The total size of the genome is 6.8 Mb and the final assembly is based on 814 Mbp of Illumina data, which provides an average 120.3x coverage of the genome.

### Genome annotation

For the general genome content description genes were identified using Prodigal [91] as part of the DOE-JGI annotation pipeline [92, 93]. The predicted CDSs were translated and used to search the National Center for Biotechnology Information (NCBI) nonredundant database, UniProt, TIGRFam, Pfam, PRIAM, KEGG, COG, and InterPro databases. The tRNAScanSE tool [94] was used to find tRNA genes, whereas ribosomal RNA genes were found by searches against models of the ribosomal RNA genes built from SILVA [95]. Other non–coding RNAs such as the RNA components of the protein secretion complex and the RNase P were identified by searching the genome for the corresponding Rfam profiles using INFERNAL (http://infernal.janelia.org). Additional gene prediction analysis and manual functional annotation was performed within the Integrated Microbial Genomes (IMG-ER) platform (http://img.jgi.doe.gov/er) [93]. The expert annotation of HMR genes was performed within the MaGe platform (https://www.genoscope.cns.fr/agc/microscope/mage) and therefore the gene numbers (CT6070v1_ XXXXXX-XX) are those from the MaGe platform. The corresponding locus tags of genes annotated in the MaGe and JGI platforms are indicated in Table S6.

### Phylogenetic analyses

Gene fragments sequences were corrected with Chromas Pro v1.33 software (Technelysium) and aligned using either ClustalX [96] or MUSCLE as implemented in MEGA, version 6 [97]. Alignments were manually edited using GeneDoc software [98]. Phylogenetic analyses were performed in MEGA6 [97] using the Neighbor-Joining method [99]. Bootstrap analysis [100] with 1000 replicates was performed to assess the support of the clusters.

### Genome analyses

The comparison of specific and common genes of symbiotic *Cupriavidus* species, presented in a Venn diagram (Fig. 2), was performed using the “Gene Phyloprofile” tool in the Microscope MaGe platform (https://www.genoscope.cns.fr/agc/microscope/mage). The orthologous counterparts in the genomes were detected by applying parameters of a minimum of 30% for protein sequences identity over a minimum of 80% of the protein length (> 30% protein MinLrap 0.8). The homologous genes were then removed from the resulting list. Transport systems were identified using the TransAAP tool [50] (TransportDB website (http://www.membranetransport.org/)) for prediction of efflux systems and transporter families.

Two methods were used for the comparison of average nucleotide identities (ANI): ANIg [42] and ANIb [43]. In order to perform the alignments using progressive Mauve software [51], the scaffolds of each draft genome (STM 6070, STM 6018, UYPR2.512 and AMP6) were firstly reordered using Mauve software on the basis of the *C. taiwanensis* LMG 19424^T^ concatenated genome. Then, reordered genomes were used to perform the alignment with progressiveMauve. Circular views by BlastAtlas were performed using the CGview server hosted at Stothard Research Group (http://stothard.afns.ualberta.ca/cgview_server/), for alignment of the STM 6070 sequence, aligned firstly on the LMG 19424^T^ concatenated sequence (CHR1, pSym, CHR2).

The essential amino acid residues that form the proximal and distal heavy-metal-binding sites have been identified for the CH34^T^ zinc-specific RND HME3a transmembrane transporter ZneA [64]. Using the ZneA protein as a backbone, we aligned the eight STM 6070 RND transmembrane proteins with those used for phylogenetic analysis (Fig. 4), to compare and identify the corresponding essential amino acid residues that form the putative proximal and distal heavy-metal-binding sites in these transporters (Table S7). In addition, we used the MaGe Microscope annotation platform [47] to analyse the syntenic arrangements of the eight HME-RND efflux systems present in STM 6070 and compare them with the HME-RND efflux systems found within six other *Cupriavidus* strains, as outlined below.

## Supplementary information

**Additional file 1: Figure S1.** Images of *Cupriavidus neocaledonicus* STM 6070 using scanning (Left) and transmission (Centre) electron microscopy and the appearance of colony morphology on solid media (Right). Images depicted here were imaged by the authors.

**Additional file 2: Figure S2.** Bacterial growth in 284 Tris-medium, in absence (○) and in presence of NiSO4 (◆: 5 mM, ●: 10 mM, ✱: 15 mM). STM 6070, studied isolate; CH34, *Cupriavidus metallidurans* CH34; AE104, plasmid cured derivative of C. *metallidurans* CH34.

**Additional file 3: Figure S3.** Tolerance to copper of symbiotic *Cupriavidus* strains STM 6070, 6018 and LMG 19424 T, performed in 284 Tris-culture medium (Mergeay et al., 1985) with 0, 0.3, 0.6 and 1.0 mM of Cu (NO3)2 concentrations.

**Additional file 4: Figure S4.** Phylogenetic tree showing the relationship of *Cupriavidus* sp. STM 6070 (shown in bold print) to other members of the order Burkholderiales based on aligned sequences of the 16S rRNA gene (1290 bp internal region). All sites were informative and there were no gap-containing sites. Phylogenetic analyses were performed using MEGA, version 6 [97]. The tree was built using the Neighbor-Joining method [99]. Bootstrap analysis [100] with 1000 replicates was performed to assess the support of the clusters. Type strains are indicated with a superscript T, strains with available genomes are indicated with *, and symbiotic *Cupriavidus* strains are indicated with blue asterisks. Brackets after the strain name contain a DNA database accession number. For LMG 19424, STM 6018, STM 6070, AMP6, JMP134, H16, N-1 and CH34CH34T the 16S rRNA sequences were recovered from sequenced genomes. Published genomes are indicated with an asterisk.

**Additional file 5: Figure S5.** Circular representation of symbiotic *Cupriavidus* genomes (by BlastAtlas using the CGview) aligned to the STM 6070 genome. The STM 6070 contigs were first aligned to the three replicons of LMG 19424^T^ Chr1/pSym/Chromid. Circles, from inside out, show GC skew (purple), GC content (black) and genomes of (1, dark grey) STM 6070; (2, green) LMG 19424^T^; (3, green) STM 6018; (4, orange) AMP6; (5, purple) UYPR2.512 and (6, red) UYMMa02A. The HME clusters A to L are marked with corresponding letters. Triangles with black borders represent clusters unique to STM 6070 and triangles without borders represent general HMR clusters.

**Additional file 6: Table S1.** General attributes and Minimum Information for the Genome Sequence (MIGS) of *Cupriavidus* strain STM 6070.

**Additional file 7: Table S2.** Number of protein coding genes of STM 6070 associated with the general COG functional categories.

**Additional file 8: Table S3.** *Cupriavidus* strains compared in this study (as bacterial isolates and/or sequenced genomes).

**Additional file 9: Table S4.** Percentage of average nucleotide identities (ANI) for ANIb and ANIg (in brackets), and percentage of conserved DNA (in bold) among *Cupriavidus* and *Ralstonia* genomes.

**Additional file 10: Table S5.** Comparison of TransAAP identified transporter genes in the genomes of *Cupriavidus neocaledonicus* STM 6070 and other *Cupriavidus* species.

**Additional file 11: Table S6.** HME determinants in STM 6070 genome and their comparison with those detected in *Cupriavidus* species.

**Additional file 12: Figure S7.** A comparison of proximal and distal sites in *Cupriavidus neocaledonicus* STM 6070 RND-HME proteins compared to related proteins in other bacterial strains.

## Data Availability

The datasets generated and/or analysed during the current study are available in the NCBI repository under bioproject 165313, [https://www.ncbi.nlm.nih.gov/bioproject/?term=165313&utm_source=gquery&utm_medium=search]; the NCBI Sequence Read Archive [https://www.ncbi.nlm.nih.gov/sra?linkname=bioproject_sra_all&from_uid=165313]; the NCBI sequence data [https://www.ncbi.nlm.nih.gov/nuccore?term=165313%5BBioProject%5D]; and the IMG repository [https://jgi.doe.gov/data-and-tools/img/] under the accession number 2513237165. All protein sequences are available in the above databases and can also be accessed through Uniprot [https://www.uniprot.org/].

## Abbreviations

½LA: Half strength Lupin Agar
ANI: Average nucleotide identity
GEBA-RNB: Genomic encyclopedia for bacteria and archaea-root nodule bacteria
HGT: Horizontal gene transfer
HME: Heavy metal efflux
HMR: Heavy metal resistance
IMG: Integrated microbial genomes
IS: Insertion sequence
