# Supplementary figures and images for: Novel heavy metal resistance gene clusters are present in the genome of Cupriavidus neocaledonicus STM 6070, a new species of Mimosa pudica microsymbiont isolated from heavy-metal-rich mining site soil
Source: BMC Genomics. 2020 Mar 6;21:214. doi: 10.1186/s12864-020-6623-z (PMC7060636; doi:10.1186/s12864-020-6623-z)

## Slide 1
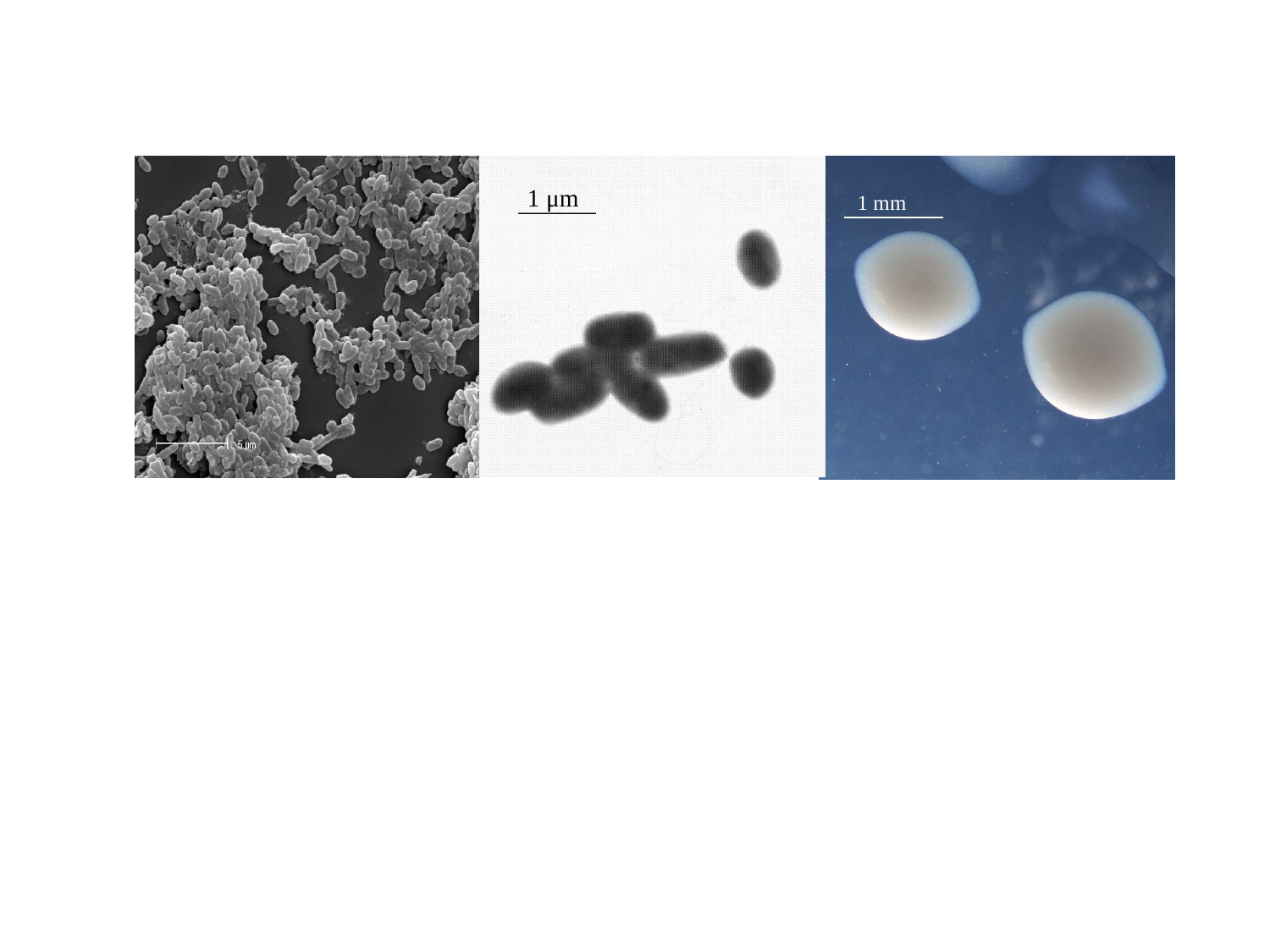

1 μm
1 mm

Supplement: Supplementary file 1 — Additional file 1: Figure S1. Images of Cupriavidus neocaledonicus STM 6070 using scanning (Left) and transmission (Centre) electron microscopy and the appearance of colony morphology on solid media (Right). Images depicted here were imaged by the authors. [file 12864_2020_6623_MOESM1_ESM.pptx]

## Slide 1
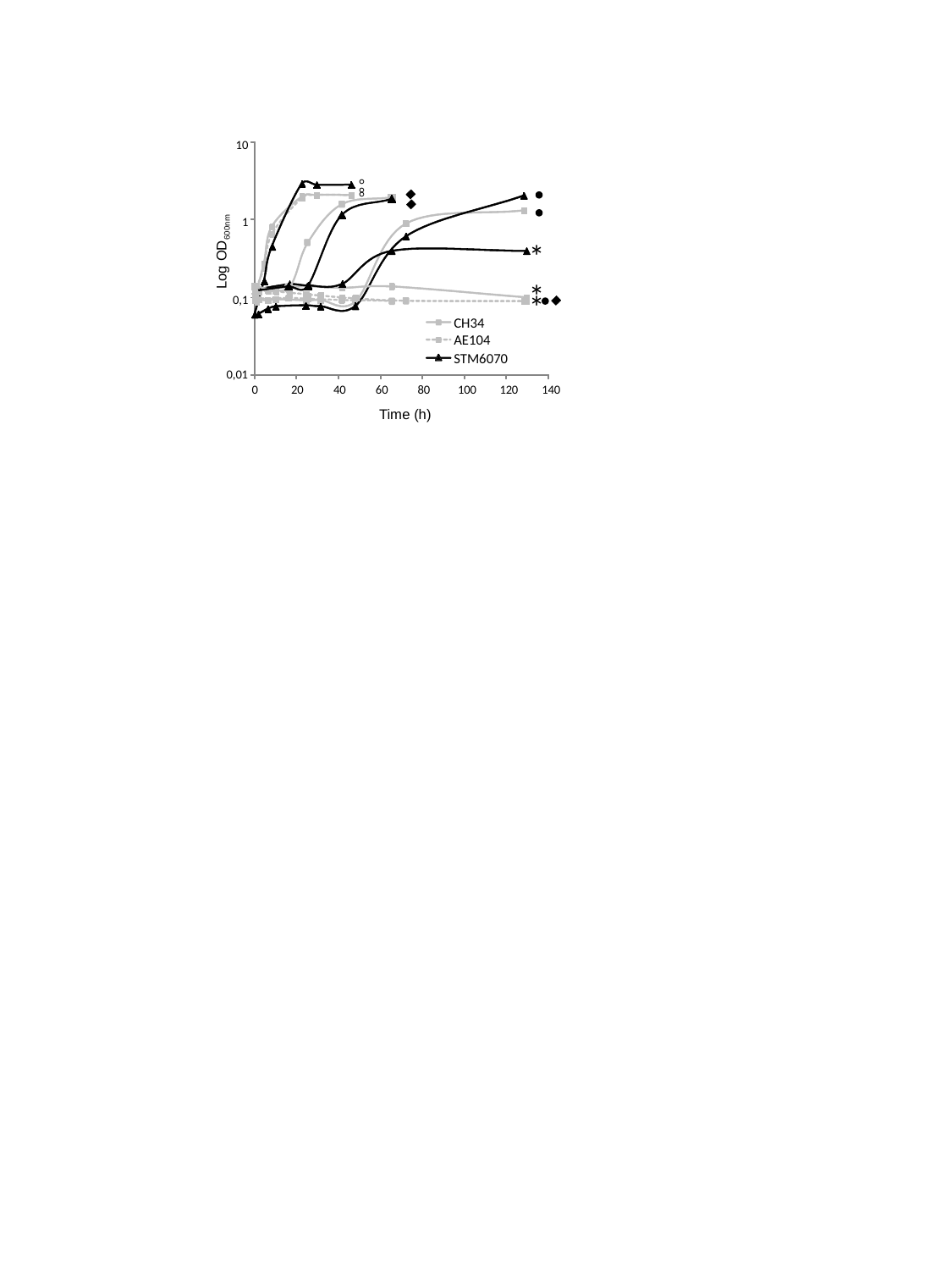

10



1
*
Log OD600nm
*
*
0,1
CH34
AE104
STM6070
0,01
0
20
40
60
80
100
120
140
Time (h)

Supplement: Supplementary file 2 — Additional file 2: Figure S2. Bacterial growth in 284 Tris-medium, in absence (○) and in presence of NiSO4 (◆: 5 mM, ●: 10 mM, ✱: 15 mM). STM 6070, studied isolate; CH34, Cupriavidus metallidurans CH34; AE104, plasmid cured derivative of C. metallidurans CH34. [file 12864_2020_6623_MOESM2_ESM.pptx]

## Slide 1
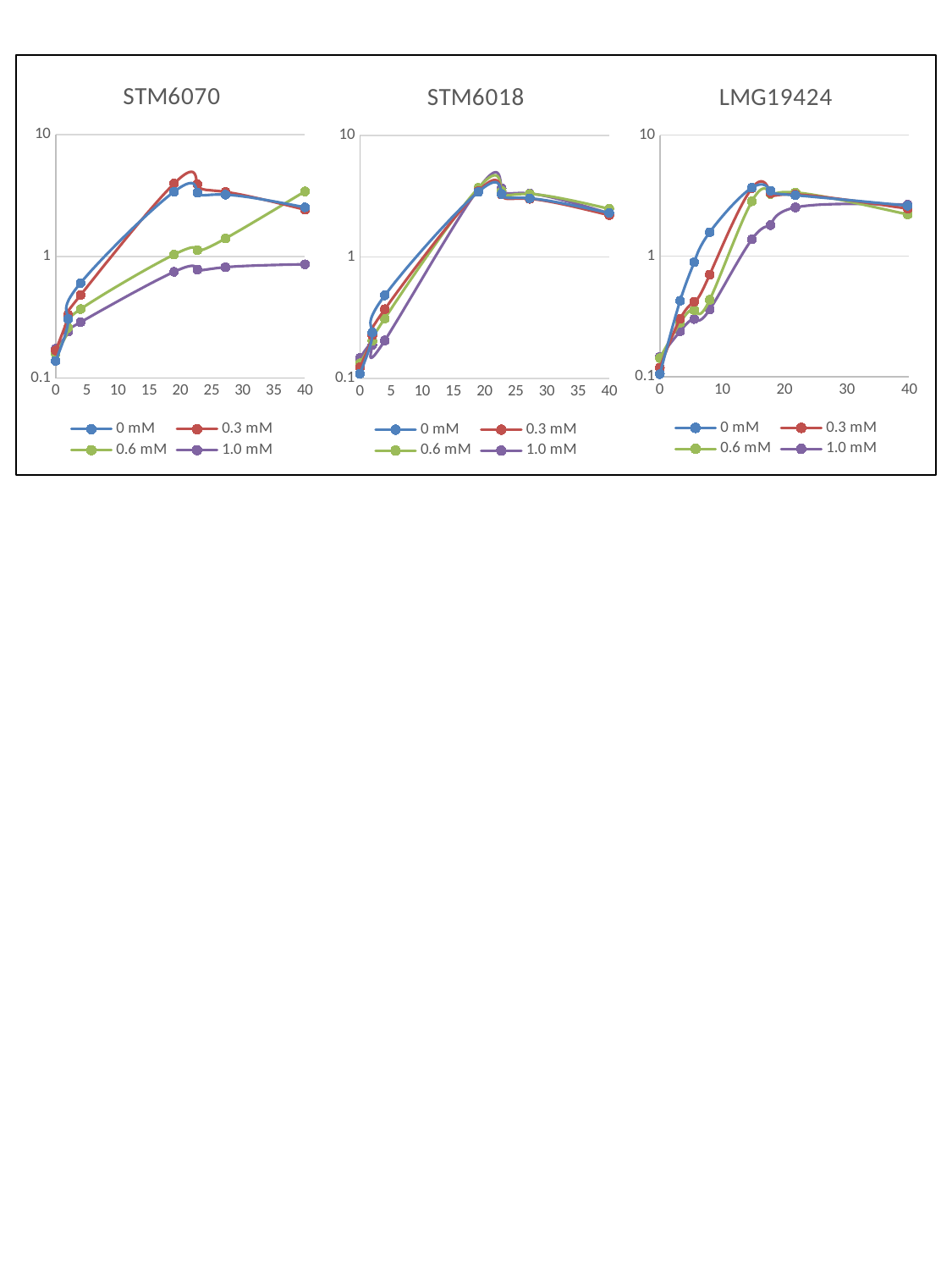

### Chart: STM6070
| Category | 0 mM | 0.3 mM | 0.6 mM | 1.0 mM |
|---|---|---|---|---|
### Chart: LMG19424
| Category | 0 mM | 0.3 mM | 0.6 mM | 1.0 mM |
|---|---|---|---|---|
### Chart: STM6018
| Category | 0 mM | 0.3 mM | 0.6 mM | 1.0 mM |
|---|---|---|---|---|

Supplement: Supplementary file 3 — Additional file 3: Figure S3. Tolerance to copper of symbiotic Cupriavidus strains STM 6070, 6018 and LMG 19424 T, performed in 284 Tris-culture medium (Mergeay et al., 1985) with 0, 0.3, 0.6 and 1.0 mM of Cu (NO3)2 concentrations. [file 12864_2020_6623_MOESM3_ESM.pptx]

## Slide 1
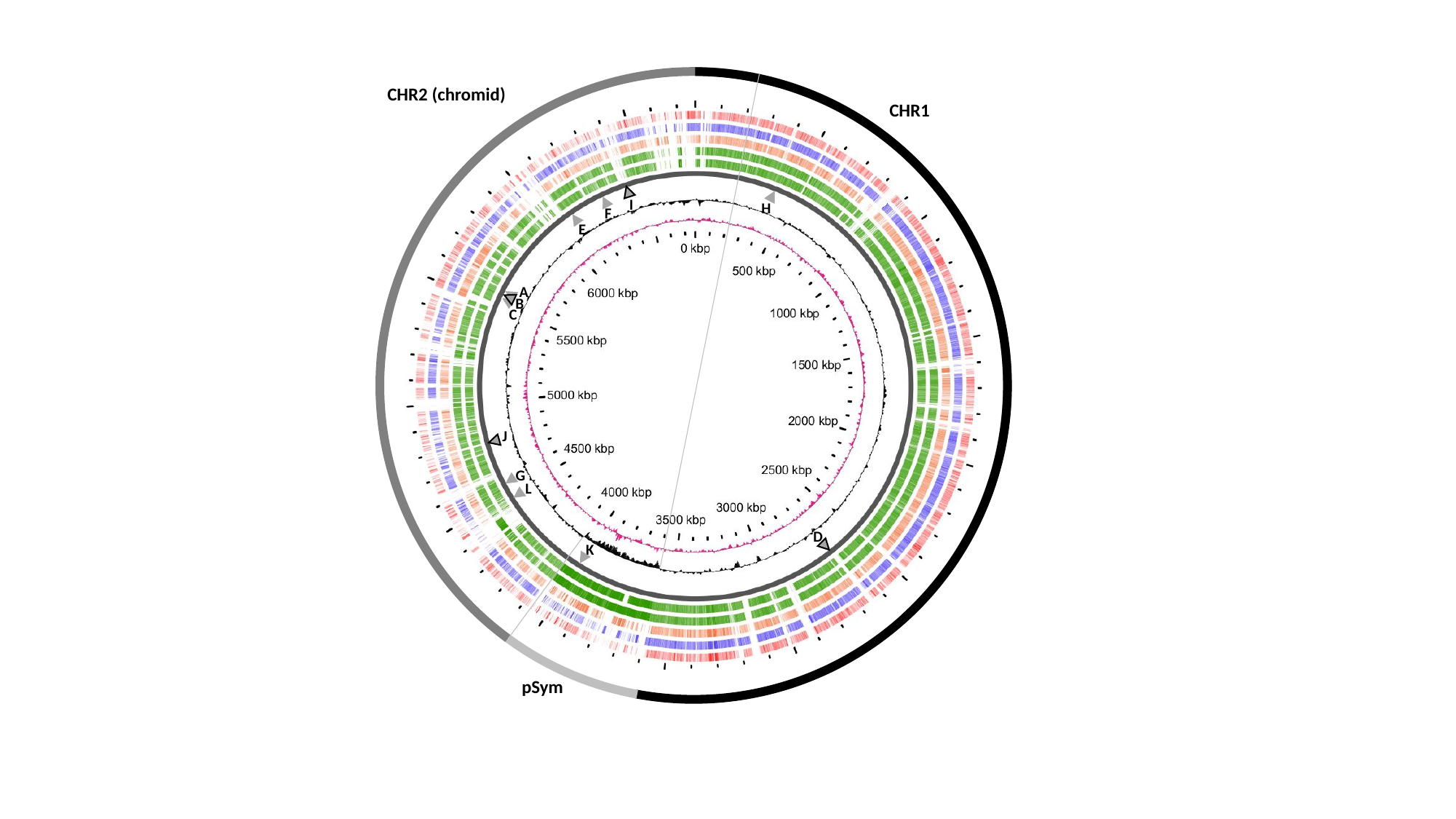

CHR2 (chromid)
CHR1
I
H
F
E
A
B
C
J
G
L
D
K
pSym

Supplement: Supplementary file 5 — Additional file 5: Figure S5. Circular representation of symbiotic Cupriavidus genomes (by BlastAtlas using the CGview) aligned to the STM 6070 genome. The STM 6070 contigs were first aligned to the three replicons of LMG 19424T Chr1/pSym/Chromid. Circles, from inside out, show GC skew (purple), GC content (black) and genomes of (1, dark grey) STM 6070; (2, green) LMG 19424T; (3, green) STM 6018; (4, orange) AMP6; (5, purple) UYPR2.512 and (6, red) UYMMa02A. The HME clusters A to L are marked with corresponding letters. Triangles with black borders represent clusters unique to STM 6070 and triangles without borders represent general HMR clusters. [file 12864_2020_6623_MOESM5_ESM.pptx]
