# Supplementary material for: Novel heavy metal resistance gene clusters are present in the genome of Cupriavidus neocaledonicus STM 6070, a new species of Mimosa pudica microsymbiont isolated from heavy-metal-rich mining site soil
Source: BMC Genomics. 2020 Mar 6;21:214. doi: 10.1186/s12864-020-6623-z (PMC7060636; doi:10.1186/s12864-020-6623-z)
Supplement: Supplementary file 4 — Additional file 4: Figure S4. Phylogenetic tree showing the relationship of Cupriavidus sp. STM 6070 (shown in bold print) to other members of the order Burkholderiales based on aligned sequences of the 16S rRNA gene (1290 bp internal region). All sites were informative and there were no gap-containing sites. Phylogenetic analyses were performed using MEGA, version 6 [97]. The tree was built using the Neighbor-Joining method [99]. Bootstrap analysis [100] with 1000 replicates was performed to assess the support of the clusters. Type strains are indicated with a superscript T, strains with available genomes are indicated with *, and symbiotic Cupriavidus strains are indicated with blue asterisks. Brackets after the strain name contain a DNA database accession number. For LMG 19424, STM 6018, STM 6070, AMP6, JMP134, H16, N-1 and CH34CH34T the 16S rRNA sequences were recovered from sequenced genomes. Published genomes are indicated with an asterisk. [file 12864_2020_6623_MOESM4_ESM.pptx]

## Slide 1
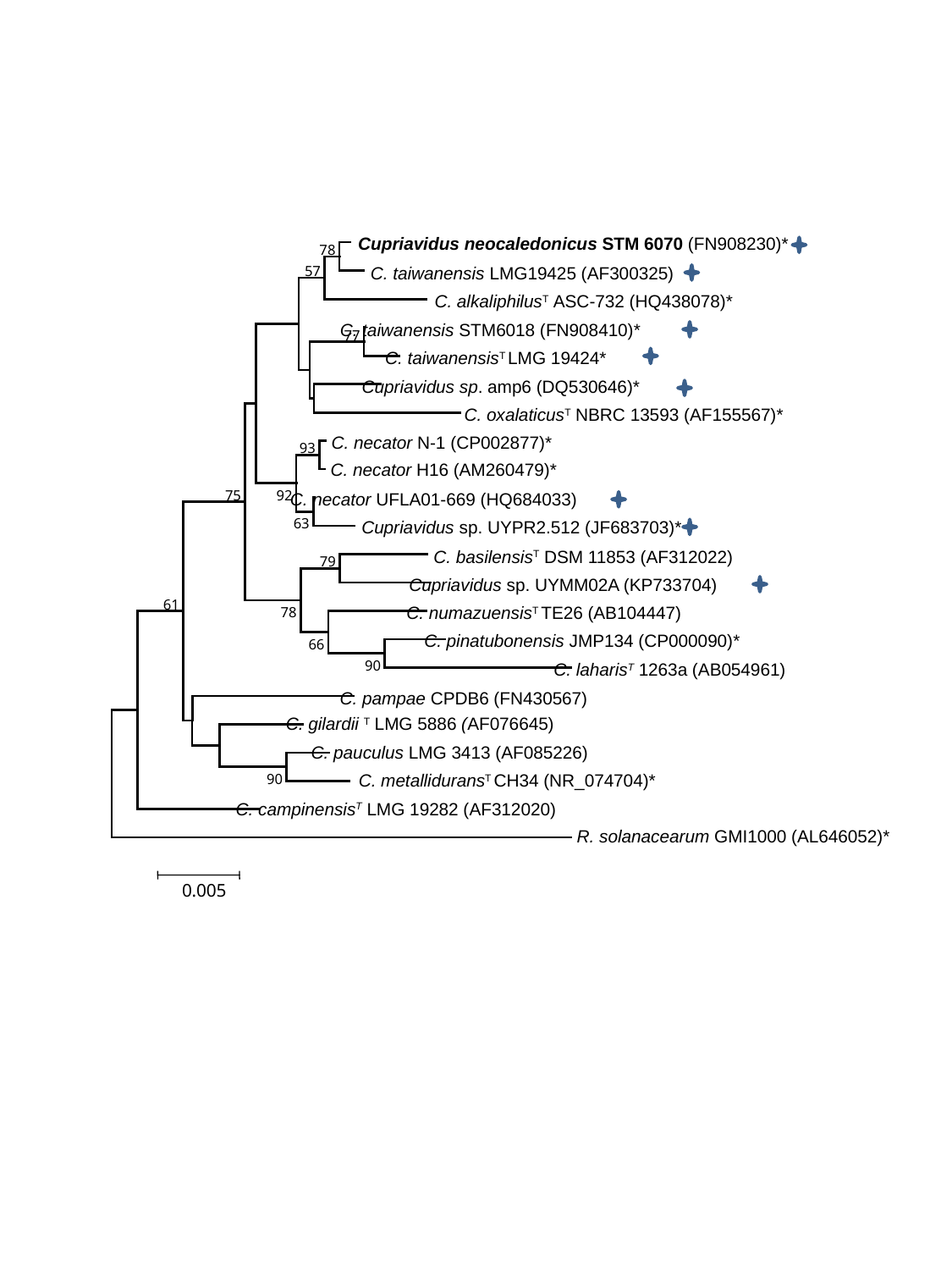

Cupriavidus neocaledonicus STM 6070 (FN908230)*
78
 C. taiwanensis LMG19425 (AF300325)
57
 C. alkaliphilusT ASC-732 (HQ438078)*
C. taiwanensis STM6018 (FN908410)*
77
 C. taiwanensisT LMG 19424*
 Cupriavidus sp. amp6 (DQ530646)*
C. oxalaticusT NBRC 13593 (AF155567)*
 C. necator N-1 (CP002877)*
93
 C. necator H16 (AM260479)*
75
92
C. necator UFLA01-669 (HQ684033)
63
 Cupriavidus sp. UYPR2.512 (JF683703)*
C. basilensisT DSM 11853 (AF312022)
79
Cupriavidus sp. UYMM02A (KP733704)
61
C. numazuensisT TE26 (AB104447)
78
 C. pinatubonensis JMP134 (CP000090)*
66
90
C. laharisT 1263a (AB054961)
C. pampae CPDB6 (FN430567)
 C. gilardii T LMG 5886 (AF076645)
 C. pauculus LMG 3413 (AF085226)
C. metalliduransT CH34 (NR_074704)*
90
 C. campinensisT LMG 19282 (AF312020)
 R. solanacearum GMI1000 (AL646052)*
0.005
