# Supplementary material for: Novel heavy metal resistance gene clusters are present in the genome of Cupriavidus neocaledonicus STM 6070, a new species of Mimosa pudica microsymbiont isolated from heavy-metal-rich mining site soil
Source: BMC Genomics. 2020 Mar 6;21:214. doi: 10.1186/s12864-020-6623-z (PMC7060636; doi:10.1186/s12864-020-6623-z)
Supplement: Supplementary file 6 — Additional file 6: Table S1. General attributes and Minimum Information for the Genome Sequence (MIGS) of Cupriavidus strain STM 6070. [file 12864_2020_6623_MOESM6_ESM.docx]

**Table S1.** General attributes and Minimum Information for the Genome Sequence (MIGS) of *Cupriavidus* strain STM 6070

| **MIGS ID** | **Property** | **Term** | **Evidence code^a^** |
| --- | --- | --- | --- |
|  | Current classification | Domain Bacteria | TAS [1] |
|  |  | Phylum *Proteobacteria* | TAS [2] |
|  |  | Class *Betaproteobacteria* | TAS [3] |
|  |  | Order *Burkholderiales* | TAS [4] |
|  |  | Family *Burkholderiaceae* | TAS [5] |
|  |  | Genus *Cupriavidus* | TAS [6] |
|  |  | Species *Cupriavidus neocaledonicus* | IDA |
|  |  | Strain: STM 6070 |  |
|  | Gram stain | Negative | TAS [7] |
|  | Cell shape | Rod | IDA |
|  | Motility | Motile | IDA |
|  | Sporulation | Non-sporulating | NAS |
|  | Temperature range | Mesophile | NAS |
|  | Optimum temperature | 28°C | TAS [7] |
| MIGS 1 | INSDC |  |  |
| MIGS 3 | Project name | GEBA-RNB | TAS [7] |
| MIGS-4 | Geographic location | Proximity to Koniambo massif, New Caledonia, France | NAS |
| MIGS-4.1 | Latitude | -21.014689 | TAS [7] |
| MIGS-4.2 | Longitude | -52.312778 | TAS [7] |
| MIGS-4.3 | Depth | 0-10 cm | NAS |
| MIGS-4.4 | Altitude | 196 m | TAS [7] |
| MIGS-5 | Sample collection | 2009 | TAS [7] |
|  |  |  |  |
| MIGS-6 | Habitat | Soil, root nodule on host | TAS [7] |
| MIGS-13 | Source Material Identifier  Project relevance | STM 6070  Symbiotic N_2_ fixation, agriculture | NAS |
| MIGS-14 | Pathogenicity | Biosafety level 1 | TAS [8] |
| MIGS-15 | Biotic relationship | Free-living/symbiotic | TAS [7] |
| MIGS-16 | Specific host | *Mimosa pudica* | TAS [7] |
| MIGS-22 | Oxygen requirement | Aerobic | TAS [7] |
| MIGS-23 | Isolation and growth conditions | YMA, TY, LB |  |
| MIGS 27 | Nucleic acid preparation | CTAB method |  |
| MIGS-28 | Libraries used | 1x Illumina Std PE library (2 x 150) |  |
| MIGS-29 | Sequencing platforms | Illumina HiSeq 2000 |  |
| MIGS 30 | Assemblers | Velvet version 1.1.04; Allpaths-LG version r39750 |  |
| MIGS-31 | Finishing strategy | Improved high-quality draft |  |
| MIGS-31.2 | Sequencing coverage | Illumina : 120.3x |  |
| MIGS 32 | Gene calling method | Prodigal 1.4 |  |
|  | Locus Tag | A3AG |  |
|  | GenBank ID | AQUR00000000 |  |
|  | GenBank Date of Release | April 19, 2013 |  |
|  | GOLD ID | Gp0009786 | [9] |
|  | NCBI BIOPROJECT ID | 165313 |  |
|  | Database: IMG | 2513237165 |  |

Evidence codes – IDA: Inferred from Direct Assay; TAS: Traceable Author Statement (i.e., a direct report exists in the literature); NAS: Non-traceable Author Statement (i.e., not directly observed for the living, isolated sample, but based on a generally accepted property for the species, or anecdotal evidence). These evidence codes are from http://www.geneontology.org/GO.evidence.shtml of the Gene Ontology project [10].

1. Woese CR, Kandler O, Wheelis ML. Towards a natural system of organisms: proposal for the domains Archaea, Bacteria, and Eucarya. Proc Natl Acad Sci U S A. 1990;87(12):4576-9.

2. Garrity GM, Bell JA, Lilburn T. Phylum XIV. Proteobacteria phyl. nov. In: Garrity GM, Brenner DJ, Kreig NR, Staley JT, editors. Bergey's Manual of Systematic Bacteriology. Second ed.: New York: Springer - Verlag; 2005. p. 1.

3. Garrity GM, Bell JA, Lilburn TE. Class II. Betaproteobacteria. In: Garrity GM, Brenner DJ, Krieg NR, Staley JT, editors. Bergey's Manual of Systematic Bacteriology. Second ed. New York: Springer - Verlag; 2005.

4. Garrity GM, Bell JA, Lilburn TE. Order 1. *Burkholderiales*. In: Garrity GM, Brenner DJ, Krieg NR, Staley JT, editors. Bergey's Manual of Systematic Bacteriology. Second ed. New York: Springer - Verlag; 2005.

5. Garrity GM, Bell JA, Lilburn TE. Family I. Burkholderiaceae. In: Garrity GM, Brenner DJ, Krieg NR, Staley JT, editors. Bergey's Manual of Systematic Bacteriology. Second ed. New York: Springer - Verlag; 2005.

6. Vandamme P, Coenye T. Taxonomy of the genus *Cupriavidus*: a tale of lost and found. Int J Syst Evol Microbiol. 2004;54:2285-9.

7. Klonowska A, Chaintreuil C, Tisseyre P, Miché L, Melkonian R, Ducousso M et al. Biodiversity of *Mimosa* *pudica* rhizobial symbionts (*Cupriavidus taiwanensis*, *Rhizobium mesoamericanum*) in New Caledonia and their adaptation to heavy metal-rich soils. FEMS Microbiol Ecol. 2012;81:618-35.

8. Biological Agents: Technical rules for biological agents. TRBA. (<http://www.baua.de/en/Topics-from-A-to-Z/Biological-Agents/TRBA/TRBA.html):466>.

9. Mukherjee S, Stamatis D, Bertsch J, Ovchinnikova G, Verezemska O, Isbandi M et al. Genomes OnLine Database (GOLD) v.6: data updates and feature enhancements. Nucleic Acids Res. 2017;45(D1):D446-D56.

10. Ashburner M, Ball CA, Blake JA, Botstein D, Butler H, Cherry JM et al. Gene Ontology: Tool for the unification of biology. The Gene Ontology Consortium. Nature Genet. 2000;25(1):25-9.
