# Supplementary material for: Novel heavy metal resistance gene clusters are present in the genome of Cupriavidus neocaledonicus STM 6070, a new species of Mimosa pudica microsymbiont isolated from heavy-metal-rich mining site soil
Source: BMC Genomics. 2020 Mar 6;21:214. doi: 10.1186/s12864-020-6623-z (PMC7060636; doi:10.1186/s12864-020-6623-z)
Supplement: Supplementary file 7 — Additional file 7: Table S2. Number of protein coding genes of STM 6070 associated with the general COG functional categories. [file 12864_2020_6623_MOESM7_ESM.docx]

Table S2. Number of protein coding genes of STM 6070 associated with the general COG functional categories.

| **Code** | **COG Category with extra row at the beginning** | **Gene Count** | **% of total (5,705)** |
| --- | --- | --- | --- |
|  | CELLULAR PROCESSES AND SIGNALING |  |  |
| D | Cell cycle control, cell division, chromosome partitioning | 33 | 0.64 |
| M | Cell wall/membrane/envelope biogenesis | 293 | 5.69 |
| N | Cell motility | 120 | 2.33 |
| O | Posttranslational modification, protein turnover, chaperones | 167 | 3.24 |
| T | Signal transduction mechanisms | 243 | 4.72 |
| U | Intracellular trafficking, secretion, and vesicular transport | 97 | 1.88 |
| V | Defense mechanisms | 121 | 2.35 |
| W | Extracellular structures | 52 | 1.01 |
| Z | Cytoskeleton | 1 | 0.02 |
|  | INFORMATION STORAGE AND PROCESSING |  |  |
| A | RNA processing and modification | 1 | 0.02 |
| B | Chromatin structure and dynamics | 3 | 0.06 |
| J | Translation, ribosomal, structure and biogenesis | 228 | 4.43 |
| K | Transcription | 497 | 9.66 |
| L | Replication, recombination and repair | 138 | 2.68 |
|  | METABOLISM |  |  |
| C | Energy production and conversion | 503 | 9.77 |
| E | Amino acid transport and metabolism | 474 | 9.21 |
| F | Nucleotide transport and metabolism | 96 | 1.87 |
| G | Carbohydrate transport and metabolism | 239 | 4.64 |
| H | Coenzyme transport and metabolism | 237 | 4.60 |
| I | Lipid transport and metabolism | 354 | 6.88 |
| P | Inorganic ion transport and metabolism | 290 | 5.63 |
| Q | Secondary metabolite biosynthesis, transport and catabolism | 201 | 3.91 |
|  |  |  |  |
|  | POORLY CHARACTERIZED |  |  |
| R | General function prediction only | 504 | 9.79 |
| S | Function unknown | 216 | 4.20 |
| X | Phage-derived proteins, transposases and other mobilome components | 39 | 0.76 |
|  | Not in COGs | 1,682 | 27.21 |
