# Supplementary material for: Novel heavy metal resistance gene clusters are present in the genome of Cupriavidus neocaledonicus STM 6070, a new species of Mimosa pudica microsymbiont isolated from heavy-metal-rich mining site soil
Source: BMC Genomics. 2020 Mar 6;21:214. doi: 10.1186/s12864-020-6623-z (PMC7060636; doi:10.1186/s12864-020-6623-z)
Supplement: Supplementary file 8 — Additional file 8: Table S3. Cupriavidus strains compared in this study (as bacterial isolates and/or sequenced genomes). [file 12864_2020_6623_MOESM8_ESM.docx]

Table S3. *Cupriavidus* strains compared in this study (as bacterial isolates and/or sequenced genomes).

| **Bacterium** | **Isolation origin** | **Isolation place** | **reference** |
| --- | --- | --- | --- |
| *C. alkaliphilus* ASC-732^T^ | Agave rhizosphere | San Carlos, Mexico | [1, 2] |
| *C. metallidurans* CH34^T^ | Decantation tank, zinc factory | Belgium | [3, 4] |
| *C. metallidurans* AE104 | CH34^T^ devoid of the plasmids pMOL28 and pMOL30 | - | [5] |
| *C. necator* H16 | healthy small pond | - | [6] |
| *C. necator* N1^T^ | Soil | Pennsylvania, USA | [7, 8] |
| ***Cupriavidus* *neocaledonicus* STM 6070** | Nodule of *Mimosa pudica* | New Caledonia | [9] |
| *C. oxalaticus* NBRC 13593^T^ | Earthworm | India | [10, 11] |
| *C. pinatubonensis* JMP134 | Soil | Australia | [12, 13] |
| *C. taiwanensis* LMG19424^T^ | Nodule of *M. pudica* | Taiwan | [14, 15] |
| *C. taiwanensis* sp. STM 6018 | Nodule of *M. pudica* | French Guiana | [16] |
| *Cupriavidus* sp. AMP6 | Nodule of *Mimosa asperata* | Texas, USA | [17] |
| *Cupriavidus* sp. UYMMa02A | Nodule of *Mimosa magenta* | Uruguay | [18] |
| *Cupriavidus* sp. UYPR2.512 | Nodule of *Parapiptadenia rigida* | Uruguay | [19] |

References

1. Estrada-de los Santos P, Vacaseydel-Aceves NB, Martínez-Aguilar L, Cruz-Hernández MA, Mendoza-Herrera A, Caballero-Mellado J. *Cupriavidus* and *Burkholderia* species associated with agricultural plants that grow in alkaline soils. J Microbiol. 2011;49(6):867-76.

2. Estrada-de los Santos P, Martínez-Aguilar L, López-Lara IM, Caballero-Mellado J. *Cupriavidus alkaliphilus* sp nov., a new species associated with agricultural plants that grow in alkaline soils. Syst Appl Microbiol. 2012;35(5):310-4.

3. Mergeay M, Nies D, Schlegel HG, Gerits J, Charles P, Van Gijsegem F. *Alcaligenes eutrophus* CH34 is a facultative chemolithotroph with plasmid-bound resistance to heavy metals. J Bacteriol. 1985;162(1):328-34.

4. Janssen PJ, Van Houdt R, Moors H, Monsieurs P, Morin N, Michaux A et al. The complete genome sequence of *Cupriavidus metallidurans* strain CH34, a master survivalist in harsh and anthropogenic environments. PLoS One. 2010;5(5).

5. Monchy S, Benotmane MA, Janssen P, Vallaeys T, Taghavi S, van der Lelie D et al. Plasmids pMOL28 and pMOL30 of *Cupriavidus metallidurans* are specialized in the maximal viable response to heavy metals. J Bacteriol. 2007;189(20):7417-25.

6. Pohlmann A, Fricke WF, Reinecke F, Kusian B, Liesegang H, Cramm R et al. Genome sequence of the bioplastic-producing "Knallgas" bacterium *Ralstonia eutropha* H16. Nature Biotechnol. 2006;24(10):1257-62.

7. Makkar NS, Casida LE. *Cupriavidus necator* gen. nov., sp. nov.: a nonobligate bacterial predator of bacteria in soil. Int J Syst Bacteriol. 1987;37(4):323-6.

8. Poehlein A, Kusian B, Friedrich B, Daniel R, Bowien B. Complete genome sequence of the type strain *Cupriavidus necator* N-1. J Bacteriol. 2011;193(18):5017-.

9. Klonowska A, Chaintreuil C, Tisseyre P, Miché L, Melkonian R, Ducousso M et al. Biodiversity of *Mimosa* *pudica* rhizobial symbionts (*Cupriavidus taiwanensis*, *Rhizobium mesoamericanum*) in New Caledonia and their adaptation to heavy metal-rich soils. FEMS Microbiol Ecol. 2012;81:618-35.

10. Quayle JR, Taylor GA. Carbon assimilation by *Pseudomonas oxalaticus* (OXI). 5. Purification and properties of glyoxylic dehydrogenase. Biochem J. 1961;78(3):611-5.

11. Vandamme P, Coenye T. Taxonomy of the genus *Cupriavidus*: a tale of lost and found. Int J Syst Evol Microbiol. 2004;54:2285-9.

12. Don RH, Pemberton JM. Genetic and physical map of the 2,4-dichlorophenoxyacetic acid-degradative plasmid pJP4. J Bacteriol. 1985;161(1):466-8.

13. Lykidis A, Pérez-Pantoja D, Ledger T, Mavromatis K, Anderson IJ, Ivanova NN et al. The complete multipartite genome sequence of *Cupriavidus necator* JMP134, a versatile pollutant degrader. PLoS One. 2010;5(3):e9729-e.

14. Chen WM, Moulin L, Bontemps C, Vandamme P, Béna G, Boivin-Masson C. Legume symbiotic nitrogen fixation by beta-proteobacteria is widespread in nature. J Bacteriol. 2003;185:7266-672.

15. Amadou C, Pascal G, Mangenot S, Glew M, Bontemps C, Capela D et al. Genome sequence of the beta-rhizobium *Cupriavidus taiwanensis* and comparative genomics of rhizobia. Genome Res. 2008;18(9):1472-83.

16. Mishra RP, Tisseyre P, Melkonian R, Chaintreuil C, Miché L, Klonowska A et al. Genetic diversity of *Mimosa pudica* rhizobial symbionts in soils of French Guiana: investigating the origin and diversity of *Burkholderia phymatum* and other beta-rhizobia. FEMS Microbiol Ecol. 2012;79(2):487-503.

17. De Meyer SE, Parker M, Van Berkum P, Tian R, Seshadri R, Reddy TBK et al. High-quality permanent draft genome sequence of the *Mimosa asperata* nodulating *Cupriavidus* sp strain AMP6. Stand Genom Sci. 2015;10:80.

18. Iriarte A, Platero R, Romero V, Fabiano E, Sotelo-Silveira JR. Draft genome sequence of *Cupriavidus* UYMMa02A, a novel beta-rhizobium species. Genome Announc. 2016;4(6):e01258-16.

19. De Meyer SE, Fabiano E, Tian R, Van Berkum P, Seshadri R, Reddy TBK et al. High-quality permanent draft genome sequence of the *Parapiptadenia rigida* nodulating *Cupriavidus* sp strain UYPR2.512. Stand Genom Sci. 2015;10:80.
