# Supplementary material for: Novel heavy metal resistance gene clusters are present in the genome of Cupriavidus neocaledonicus STM 6070, a new species of Mimosa pudica microsymbiont isolated from heavy-metal-rich mining site soil
Source: BMC Genomics. 2020 Mar 6;21:214. doi: 10.1186/s12864-020-6623-z (PMC7060636; doi:10.1186/s12864-020-6623-z)
Supplement: Supplementary file 9 — Additional file 9: Table S4. Percentage of average nucleotide identities (ANI) for ANIb and ANIg (in brackets), and percentage of conserved DNA (in bold) among Cupriavidus and Ralstonia genomes. [file 12864_2020_6623_MOESM9_ESM.docx]

Table S4. Percentage of average nucleotide identities (ANI) for ANIb and ANIg (in brackets), and percentage of conserved DNA (in bold) among *Cupriavidus* and *Ralstonia* genomes.

| Target | LMG 19424^T^ | STM 6018 | STM 6070 | UYPR2.512 | AMP6 | NBRC 13593^T^ | N1^T^ | JMP134 | ASC-732^T^ | CH34^T^ |
| --- | --- | --- | --- | --- | --- | --- | --- | --- | --- | --- |
| Query |  |  |  |  |  |  |  |  |  |  |
| *C. taiwanensis* LMG 19424^T^ | --- | **91.89** | **80.49** |  |  |  |  |  | **77.02** |  |
| *C. taiwanensis* STM 6018 | 98.72 (99.03**)** | --- | **84.55** |  |  |  |  |  | **57.96** |  |
| *Cupriavidus neocaledonicus.* STM 6070 | 93.33 (94.6) | 93.4 (94.7) | --- |  |  |  |  |  | **76.48** |  |
| *Cupriavidus sp.* UYPR2.512 | 87.22 (89.75) | 87.23 (89.67) | 87.29 (89.62) | --- |  |  | **63.87** |  |  |  |
| *Cupriavidus sp.* AMP6 | 85.66 (88.40) | 85.71 (88.43) | 85.65 (88.35) | 85.74 (88.11) | --- | **73.38** |  |  |  |  |
| *C. oxalaticus* NBRC13593^T^ | 85.84 (88.72) | 85.81 (88.75) | 85.93 (88.84) | 86.23 (88.69) | 92.69 (94.44) | --- |  |  |  |  |
| *C. necator* N1^T^ | 86.7 (89.68) | 86.62 (89.60) | 86.46 (89.48) | 93.43 (95.27) | 85.3 (88.28) | 86.36 (88.80) | --- |  |  |  |
| *C. pinatubonensis* JMP134 | 81.36 (83.91) | 81.25 (83.84) | 81.42 (83.97) | 81.76 (84.05) | 81.48 (83.92) | 81.7 (84.12) | 81.58 (83.91) | --- |  |  |
| *C. alkaliphillus* ASC-732^T^ | 92.54 (93.90) | 92.59 (93.89) | 93.32 (94.62) | 88.18 (90.03) | 86.11 (88.42) | 86.0 (88.88) | 88.28 (90.07) | 81.31 (83.90) | --- |  |
| *C. metallidurans* CH34^T^ | 78.88 (81.60) | 78.67 (81.38) | 78.75 (81.39) | 78.71 (81.29) | 78.67 (81.34) | 78.82 (81.68) | 78.78 (81.29) | 78.45 (80.87) | 78.88 (81.48) | --- |

ANIb values were calculated with JSpecies (based on whole genome BLAST alignment) [42]. ANIg values (in brackets) were calculated using the ANI tool in IMG [43]. Species affiliation cut-off scores were >95% (over 69% of conserved DNA) for ANIb and >96.5% for ANIg. The % of conserved DNA values are shown for ANIb values higher than 90% (in bold). Values in red font reveal strains that belong to the same species. Genomes were downloaded from Genbank accessions when already published. Species compared: *Cupriavidus taiwanensis* strains LMG 19424^T^ and STM 6018; *Cupriavidus* *neocaledonicus* STM 6070; *Cupriavidus* sp. UYPR2.512; *Cupriavidus* sp. AMP6; *C. alkaliphilus* strain ASC-732^T^; *C. necator* N1^T^; *C. oxalaticus* NBRC 13593^T^; *C. pinatubonensis* JMP134; *C. metallidurans* CH34^T^.
