# Supplementary material for: Novel heavy metal resistance gene clusters are present in the genome of Cupriavidus neocaledonicus STM 6070, a new species of Mimosa pudica microsymbiont isolated from heavy-metal-rich mining site soil
Source: BMC Genomics. 2020 Mar 6;21:214. doi: 10.1186/s12864-020-6623-z (PMC7060636; doi:10.1186/s12864-020-6623-z)
Supplement: Supplementary file 10 — Additional file 10: Table S5. Comparison of TransAAP identified transporter genes in the genomes of Cupriavidus neocaledonicus STM 6070 and other Cupriavidus species. [file 12864_2020_6623_MOESM10_ESM.docx]

Table S5. Comparison of TransAAP identified transporter genes in the genomes of *Cupriavidus* *neocaledonicus* STM 6070 and other *Cupriavidus* species.

|  |  | Number of genes detected in genomes of *Cupriavidus* species^b^ | | | | | | |
| --- | --- | --- | --- | --- | --- | --- | --- | --- |
| Transporter classification | Number^a^ | Cmet  CH34^T^ | *Cnec*  H16 | *Cnec*  N-1^T^ | *Cpin* JMP134 | *Ctai*  LMG 19424^T^ | *Cneo*  STM 6070 | *Cneo*  STM 6070  HMR genes^c^ |
| MFS Superfamily | 2.A.1 | 89 | 108 | 142 | 125 | 87 | 106 | **2** |
| CDF Family | 2.A.4 | 5 | 3 | 2 | 4 | 4 | 4 | **4** |
| RND Superfamily | 2.A.6 | 26 | 22 | 28 | 22 | 18 | 31 | **8** |
| CHR Family | 2.A.51 | 5 | 2 | 4 | 4 | 2 | 3 | **2** |
| NiCoT Family | 2.A.52 | 1 | 2 | 0 | 0 | 0 | 0 | **0** |
| ACR3 Family | 2.A.59 | 1 | 1 | 1 | 1 | 2 | 2 | **2** |
| P-ATPase Superfamily | 3.A.3 | 13 | 11 | 8 | 10 | 6 | 10 | **5** |
| Total |  | 140 | 149 | 185 | 166 | 119 | 156 | **23** |

^a^Subclass of transporters as defined in [51, 52], also see in the text. ^b^*Cmet*, C. metallidurans; *Cnec, C. necator*; *Cneo*, *C. neocaledonicus; Cpin, C. pinatubonensis*; and *Ctai*, *C. taiwanensis.* Data includes information from Janssen *et al*. [35]. ^c^Genes identified in this study as encoding proteins putatively involved in metal tolerance.
